# Aberrant hepatic lipid storage and metabolism in canine portosystemic shunts

**DOI:** 10.1371/journal.pone.0186491

**Published:** 2017-10-19

**Authors:** Lindsay Van den Bossche, Vivien A. C. Schoonenberg, Iwan A. Burgener, Louis C. Penning, Ingrid M. Schrall, Hedwig S. Kruitwagen, Monique E. van Wolferen, Guy C. M. Grinwis, Anne Kummeling, Jan Rothuizen, Jeroen F. van Velzen, Nikolas Stathonikos, Martijn R. Molenaar, Bernd J. Helms, Jos F. H. M. Brouwers, Bart Spee, Frank G. van Steenbeek

**Affiliations:** 1 Department of Clinical Sciences of Companion Animals, Faculty of Veterinary Medicine, Utrecht University, Utrecht, The Netherlands; 2 Department of Pathobiology, Faculty of Veterinary Medicine, Utrecht University, Utrecht, The Netherlands; 3 Laboratory for Translational Immunology, University Medical Center Utrecht, Utrecht, The Netherlands; 4 Department of Pathology, University Medical Center, Utrecht, The Netherlands; 5 Department of Biochemistry and Cell Biology, Faculty of Veterinary Medicine & Institute of Biomembranes, Utrecht, The Netherlands; Karl-Franzens-Universitat Graz, AUSTRIA

## Abstract

Non-alcoholic fatty liver disease (NAFLD) is a poorly understood multifactorial pandemic disorder. One of the hallmarks of NAFLD, hepatic steatosis, is a common feature in canine congenital portosystemic shunts. The aim of this study was to gain detailed insight into the pathogenesis of steatosis in this large animal model. Hepatic lipid accumulation, gene-expression analysis and HPLC-MS of neutral lipids and phospholipids in extrahepatic (EHPSS) and intrahepatic portosystemic shunts (IHPSS) was compared to healthy control dogs. Liver organoids of diseased dogs and healthy control dogs were incubated with palmitic- and oleic-acid, and lipid accumulation was quantified using LD540. In histological slides of shunt livers, a 12-fold increase of lipid content was detected compared to the control dogs (EHPSS *P*<0.01; IHPSS *P* = 0.042). Involvement of lipid-related genes to steatosis in portosystemic shunting was corroborated using gene-expression profiling. Lipid analysis demonstrated different triglyceride composition and a shift towards short chain and omega-3 fatty acids in shunt versus healthy dogs, with no difference in lipid species composition between shunt types. All organoids showed a similar increase in triacylglycerols after free fatty acids enrichment. This study demonstrates that steatosis is probably secondary to canine portosystemic shunts. Unravelling the pathogenesis of this hepatic steatosis might contribute to a better understanding of steatosis in NAFLD.

## Introduction

Non-alcoholic fatty liver disease (NAFLD) is the most common liver disorder in men with an estimated prevalence ranging 25% up to 45% worldwide [1]. NAFLD includes related disorders from the earliest stage hepatic steatosis, to the more progressive stage non-alcoholic steatohepatitis, of which the latter can progress to cirrhosis and hepatic cellular carcinoma [1,2]. The pathophysiology, however, is still poorly understood and NAFLD is associated with cardiovascular disease, diabetes mellitus type 2, and chronic kidney disease [2–4]. Although murine models resemble monogenic forms of NAFLD [5], these diseases in mice are often incapable of fully mimicking the multifactorial nature of human NAFLD.

Congenital portosystemic shunts (CPSS) are vascular anomalies that connect the portal vein with the systemic circulation, causing portal blood to bypass the hepatic parenchyma [6,7]. Although extremely rare in humans [6], CPSS occur frequently in dogs and can be divided into two subtypes; extrahepatic portosystemic shunts (EHPSS) and intrahepatic portosystemic shunts (IHPSS) [8]. The absence of normal hepatic portal blood flow leads to liver atrophy, hypoplasia of the portal vein, and hepatic encephalopathy [9–11]. Histological changes observed in CPSS include hepatocellular atrophy, enlarged portal areas, periportal sinusoidal dilatation, small or not detectable portal veins, and (peri)portal arteriole proliferation. Other findings include hepatic fibrosis, bile duct proliferation, portal lymphangiectasis, and hepatocellular steatosis [11–15].

Histological evaluation of hepatic biopsies after surgical attenuation of the shunt revealed a decrease in steatosis, suggesting steatosis in CPSS could be induced by hepatic hypoxia or a disturbed fatty acid metabolism [14]. Steatosis in CPSS dogs could be explained by a genetically determined factor [7] or by altered metabolism secondary to disease processes and the resulting hepatic injury [12,14].

This study was performed to evaluate steatosis in canine congenital portosystemic shunting. As steatosis is observed histologically in both shunt types [11–15], we expect that hepatic steatosis occurs secondary to portosystemic shunting. In-depth analysis of the lipid metabolism of dogs with CPSS with gene- and lipid-profiling combined with organoid disease modelling will give insight in the pathogenesis of primary or secondary hepatic steatosis. This in-depth analysis might serve as a model for human steatosis as observed in NAFLD and lead to novel treatment methods for steatosis in human and veterinary medicine.

## Methods

### Animals and samples

Liver material was obtained from privately owned dogs with portosystemic shunts, referred to the University Clinic for Companion Animals (Department of Clinical Sciences of Companion Animals, Utrecht University). Permission was obtained from the dog owners using informed consent. CPSS was diagnosed based on increased fasting plasma ammonia levels (reference values 15–45 μmol/L) [16,17], ultrasound visualization and classification of the shunt, and finally confirmed during surgery. Fresh wedge liver biopsies were taken during the surgical attenuation of the shunt [18]. Liver tissue from healthy dogs was used as a control in this study. These dogs were euthanized for other unrelated research, data were collected according to the Act on Veterinary Practice and the procedure was approved by the local ethics committee (DEC Utrecht), as required under Dutch legislation (ID 2007.III.08.110). Liver tissue was obtained as surplus material (University 3R policy). The absence of an underlying liver disease was confirmed histologically by a board certified veterinary pathologist. The analysis of lipid accumulation by Oil-red-O staining, mRNA expression using quantitative reversed transcriptase PCR (RT-qPCR) and the profiling of neutral lipids and phospholipids was performed on overlapping hepatic tissue of dogs with a shunt (EHPSS *n* = 7 and IHPSS *n* = 5) and compared to healthy control dogs (*n* = 4). For the microarray analysis a cohort of 49 samples (EHPSS *n* = 32, IHPSS *n* = 15, and control *n* = 2) was used. Nine cases for both IHPSS and EHPSS from this analysis were replicated in qPCR and the sample set was supplemented with 46 additional samples (EHPSS *n* = 19, IHPSS *n* = 14, and control *n* = 13). Hepatic tissue of 12 dogs (EHPSS, IHPSS, and healthy controls; n = 4 per group) was used for organoid culture. An overview of the sample use and overlapping samples is given in supplementary data (S1 Fig). For Oil-red-O staining, hepatic biopsies were placed in a Tissue-Tek® cryo-molds filled with O.C.T. Compound (Sakura Finetek Europe B.V., Alphen aan den Rijn, The Netherlands) and frozen in liquid nitrogen until use. For RNA isolation liver samples were snap frozen in liquid nitrogen.

### Oil-red-O staining

Oil-red-O staining was performed as previously described [15]. The frozen samples were cut into 8-μm sections and stained for lipids using a standard Oil-red-O (Klinipath, Duiven, The Netherlands) protocol with haematoxylin counterstaining. All Oil-red-O stained sections were evaluated blind and at random by a board-certified pathologist (GCMG), using a semi-quantitative scoring system of lipid accumulation based on lipid intensity of the stainings. Intensity grading ranged from low to high using a scale from 0 to 4. For lipid intensity analysis, slides were scanned at 20× magnification as described previously [19]. Images were extracted using Aperio ImageScope v12.0.0.5039 (Aperio, Vista, CA, USA) as a TIFF file with jpeg compression. The images were resized to 10% of their original size for digital analysis. Data of ten random snapshots were collected. The RGB data of the images was converted to a 2-bit black/white image based on thresholding the color of the dye using ImageJ (NIH; http://rsb.info.nih.gov/ij/) software. An average of the black:white ratio was calculated to determine lipid intensity scores.

### Expression profiling

Previously published microarray expression data on IHPSS and EHPSS liver tissue [20], available through GEO Series accession number GSE39005 (http://www.ncbi.nlm.nih.gov/geo/query/acc.cgi?-acc=GSE39005), was used to determine the 20 most up and 20 most down regulated genes in both EHPSS and IHPSS versus control dogs. A corresponding list of lipid related genes for IHPSS and EHPSS was selected for further confirmation. Genes with log2-fold changes of more than 1.1 or less than -1.5 were selected to ensure that only robust differences were considered. Involvement of these genes in lipid metabolism, transport, or storage was determined based on Gene Ontology biological processes and literature.

Gene expression differences of 11 selected genes was confirmed using RT-qPCR on available cDNA obtained using the iScript™ cDNA synthesis kit as described by the manufacturers protocol (Bio-Rad, Veenendaal, The Netherlands). Primer design, validation, RT-qPCR conditions, and data analysis were performed as described previously [20]. Normalization was performed using four reference-genes; glyceraldehyde-3-phosphate dehydrogenase (*GAPDH*), ribosomal protein S5 (*RPS5*), heterogeneous nuclear ribonucleoprotein H (*HNRPH*), and ribosomal protein S19 (*RPS19*) [20] as required under MIQE-precise [21]. Details of the primers are listed in S1 Table.

### Analysis of neutral- and phospholipids by high-performance liquid chromatography-mass spectrometry (HPLC-MS)

Lipids were isolated from frozen tissue by the method of Bligh and Dyer [22] and separated in a neutral lipids and phospholipid fraction on a freshly prepared silica-G column (approximately 10 mg of 0.063–0.200 mm silica (Sigma-Aldrich, St Louis, MO, USA) [23]. Neutral lipids were eluted with two volumes acetone, dried under nitrogen gas and stored at −20°C. Just before HPLC-MS analysis, the neutral lipid fraction was reconstituted in methanol:chloroform (1:1 v/v) and separated on a Kinetex/HALO C8-e column (2.6 μm, 150 × 3.00 mm; Phenomenex, Torrance, CA, USA). A gradient was generated from methanol:H_2_O (5:5 v/v) and methanol:isopropanol (8:2 v/v) at a constant flow rate of 0.3 ml/min. Mass spectrometry of neutral lipids (triacylglycerols (TAGs) and cholesterol) was performed using positive mode Atmospheric Pressure Chemical Ionization (APCI) on a LTQ-XL mass spectrometer (Thermo, Waltham, MA, USA). Separation of phospholipid classes was performed as described elsewhere [24].

### Isolation of canine biliary duct fragments and culture of liver organoids

Organoids were isolated and cultured as described before [25]. In short, liver tissue was dissected mechanically and digested in DMEM medium with 1% v/v FBS (Gibco, Fischer Scientific, Landsmeer, The Netherlands) containing 0.3 mg/ml type II collagenase (Gibco) and 0.3 mg/ml dispase (Gibco) at 37°C for a total of 3–5 hours. The isolated ducts were then mixed with Matrigel (BD Biosciences, Breda, The Netherlands) and seeded. Culture medium was added after gelation of the Matrigel. Culture media was based on Advanced DMEM/F12 (Invitrogen, Bleiswijk, The Netherlands) supplemented with 2% v/v B27 (Invitrogen), 1% v/v N2 (Invitrogen), 1.25 μM N-acethylcysteine (Sigma-Aldrich), 10 nM gastrin (Sigma-Aldrich), 200 ng/ml EGF (Invitrogen), 5% v/v Rspo1 conditioned medium (the Rspo1-Fc-expressing cell line was a kind gift from Dr. Calvin J. Kuo, Stanford, CA), 100 ng/ml FGF10 (Peprotech, Tebu-bio, Heerhugowaard, The Netherlands), 10 mM nicotinamide (Sigma-Aldrich), 25 ng/ml HGF (Peprotech), 100 ng/ml Noggin (Peprotech), 30% v/v Wnt3a conditioned medium (prepared as in [26], 10 μM Y-27632 2HCl (ROCK inhibitor, Selleckchem, Bio-Connect B.V., Huissen, The Netherlands), and 0.5 μM TGFβ inhibitor (A83-01, Tocris Bioscience, Abingdon, UK) grown at 37°C with 5% CO_2_ in a humidified incubator. Organoids were split by removal from Matrigel using cold Advanced DMEM/F12, mechanical dissociation into smaller fragments, and transfer into fresh Matrigel. Passage was performed weekly at a 1:4–1:8 split ratio. Medium was changed every other day.

### Treatment of organoids with free fatty acids (FFA)

Oleic acid (C18:1) and palmitic acid (C16:0) (both from Sigma-Aldrich) were conjugated with fatty acid free bovine serum albumin (BSA) (Sigma-Aldrich), molar ratio of 5:1, to a final concentration of 10 mM. Organoids were cultured in a 12-wells plate (Greiner Bio-One B.V., Alphen aan den Rijn, The Netherlands), and treated with 0.4 mM oleate/BSA- and 0.2 mM palmitate/BSA-complexes in culture media (without Wnt3a, Y-27632, A83, EGF and Noggin) for 24 h at 37°C with 5% CO_2_ in a humidified incubator. Treatment with fatty acid free BSA alone (12% w/v) served as a control.

### Flow cytometry analysis

After a 24 h incubation with FFA, organoids were collected from the Matrigel with cold advanced DMEM/F12 (Gibco), and subsequently trypsinised with 10x Trypsin (Gibco) containing 0.5 mg/ml DNAse (Sigma). Advanced DMEM/F12 with 10% FCS was added and the cell suspension was spun at 250 g for 5 min at 4°C. Pellets were resuspended in advanced DMEM/F12. Organoids were incubated with 5 μg/ml LD540 (lipophilic dye, kindly provided by prof. Christoph Thiele, Bonn, Germany) for microscopic imaging of lipid droplets [27] in DMEM medium containing 20 μg/ml fatty acid free BSA, 10μg/ml HEPES (Gibco), and 10 μg/ml Glutamax (Gibco) for 30 min in a water bath at 37°C. Incubations without LD540 in FFA medium served as a control. Cells were washed twice with HBSS (Gibco) and cells were resuspended in HBSS containing 20 μg/ml fatty acid free BSA, 1 μg/ml HEPES and 1 μg/ml Sytox Red (Molecular probes, Thermo Fisher, Bleiswijk, The Netherlands). Cell analysis was performed on a 488-laser LSRFortessa flow cytometer (Becton Dickinson, Erembodegem, Belgium). A 540/30 nm bandpass filter was installed to measure the optimum of the LD540 emission peak. Fluorescently labelled beads (CS&T beads, Becton Dickinson) were used to check the performance and verify optical path and stream flow of the flow cytometer. Dead cells were excluded with Sytox red using a 635 nm laser with an emission spectrum of 670/30 nm.

### Whole mount imaging

For whole mount fluorescent staining canine liver organoids were carefully harvested from Matrigel and fixed in 10% v/v neutral buffered formalin (Klinipath) for 45 min on ice. Fixed organoids were incubated in 0.025 μg/μL LD540 in PBS for 1 h at room temperature. After washing, nuclei were stained with DAPI and organoids were mounted with ProLong Diamond Antifade mounting medium (Life Technologies, Thermo Fisher) and imaged using a confocal microscope (Leica SPE-II).

### Statistical analysis

Oil-red-O differences in scoring were evaluated using a Student T-test. *P* values < 0.05 were considered significant. The results of the microarray analysis were reanalyzed with updated annotations using ANOVA (R version 2.2.1/MAANOVA version 0.98) [28]. Correction for multiple testing (Permutation F2-test using 5,000 permutations) was performed and *P* < 0.05 was considered statistically significant. In RT-qPCR log-values of normalized relative expression were used to obtain a normal distribution. A Levene’s test was used to determine if the data was normally distributed. A Kruskal-Wallis test was performed to observe differences between the EHPSS, IHPSS, and control group and was performed in case of multiple group testing. Any observed differences were confirmed by a Mann-Whitney U test on independent samples. Statistical significance was obtained if *P* < 0.01. Processing of the LC-MS data of neutral lipids and phospholipids was performed with XCMS under R version 3.0.2 [29,30]. Principal component analysis (PCA) was performed with the R package ‘PCAMethods’ using the nonlinear iterative partial least squares (nipals) algorithm with pareto scaling [31]. Differences in lipid accumulation after free fatty acid incubation between the EHPSS, IHPSS, and wildtype organoids measured using flow cytometry analysis, were calculated with a Kruskall-Wallis test. Observed differences were confirmed by a Mann-Whitney U test on independent samples.

## Results

### Difference in lipid accumulation between healthy and shunts liver biopsies

**Fig 1:**
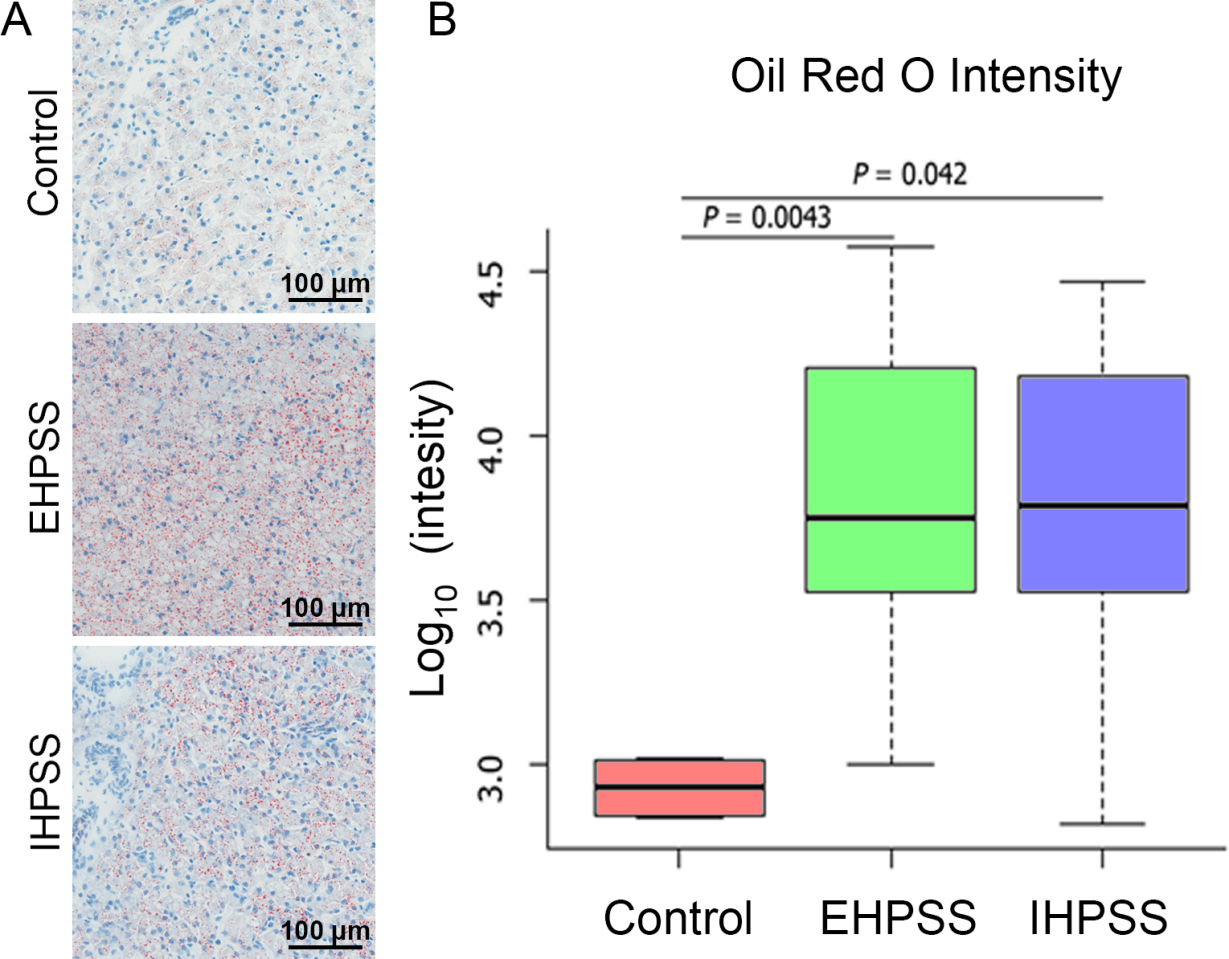
Average lipid intensity using an Oil-red-O staining in hepatic tissue of control, EHPSS and IHPSS dogs. Representative pictures of the hepatic samples from healthy dogs (*n* = 4), dogs with extrahepatic portosystemic shunts (EHPSS, *n* = 7), and intrahepatic portosystemic shunts (IHPSS, *n* = 5) are displayed left (A). The average Oil-red-O intensity is displayed in Log_10_ per sample group, representing neutral lipid staining in the observed liver samples calculated with a Students T-test and *P* < 0.05 was considered significant (B).

Oil-red-O staining for neutral lipid accumulation was increased in EHPSS and IHPSS slides compared to livers of healthy control dogs (Fig 1A). Image J quantification revealed a 12-fold increased staining intensity in both shunt types (EHPSS *P* < 0.01; IHPSS *P* < 0.05) (Fig 1B) compared to the samples of healthy control dogs in our Dutch cohort. Semi-quantitative analysis of these samples confirmed the higher hepatic neutral lipid levels in EHPSS (*P* < 0.01) and IHPSS (*P* < 0.05) compared to healthy dogs (S2 Fig).

### Similar gene-expression patterns of lipid related genes in both shunt types

**Fig 2:**
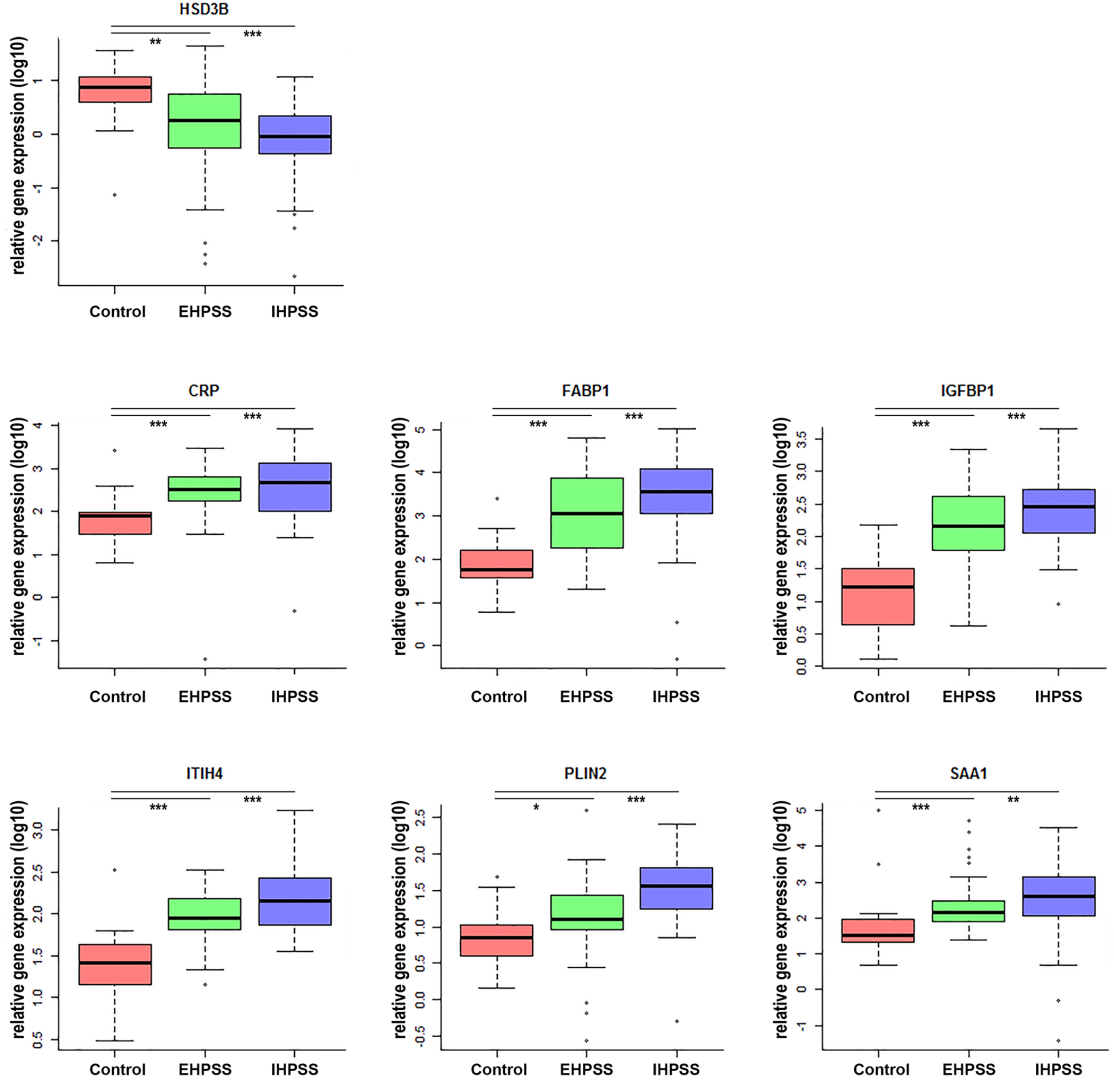
Validated lipid related genes by RT-qPCR in a validation cohort. Boxplots representing relative gene expression lipid related genes validated with RT-qPCR in a validation cohort of diseased dogs (*n* = 63). Gene expression of selected genes displayed per group: control dogs (C, *n* = 17), extrahepatic- (EH, *n* = 35), and intrahepatic portosystemic shunt (IH, *n* = 28). Significant difference between groups is presented as a line with *** (*P* ≤ 0.001), ** (0.001 < *P* ≤ 0.01), or * (0.01 < *P* < 0.05).

Data retrieved from previously published data sets revealed that similar genes were differentially expressed in both shunts compared to samples of healthy dogs in the microarray analysis (S2 Table). Interestingly 11 out of the selected top 24 differentially expressed genes are related to lipid-metabolism, -transport, or -storage emphasizing the importance of the altered lipid metabolism here. Nine gene products (*CBR2*, *CRP*, *ELOVL2*, *FABP1*, *IGFBP1*, *ITIH3*, *ITIH4*, *PLIN2*, and *SAA1*) were significantly upregulated in both shunt groups whereas *HSD3B* and *SEC14L3* were significantly downregulated compared to healthy control dogs in the microarray analysis. The expression of these 11 genes was validated by RT-qPCR in an independent cohort. For technical reasons, no RT-qPCR data could be obtained for *CBR2*. Due to difficulties in primer design for *ELOVL2* but the interest in this gene, primers were ordered for *ELOVL5* and *ELOVL6* to gain information about the *ELOVL2* pathway. The RT-qPCR analysis confirmed the microarray results for seven genes of interest (*P* < 0.01), namely *CRP*, *FABP1*, *HSD3B*, *IGFBP1*, *ITIH4*, *PLIN2*, and *SAA1* (Fig 2). *ELOVL2-pathway*, *ITIH3*, and *SEC14L3* expression levels were not significantly different in the validation cohort.

### Analysis of neutral- and phospholipids reveal similar lipid species in both shunt types

**Fig 3:**
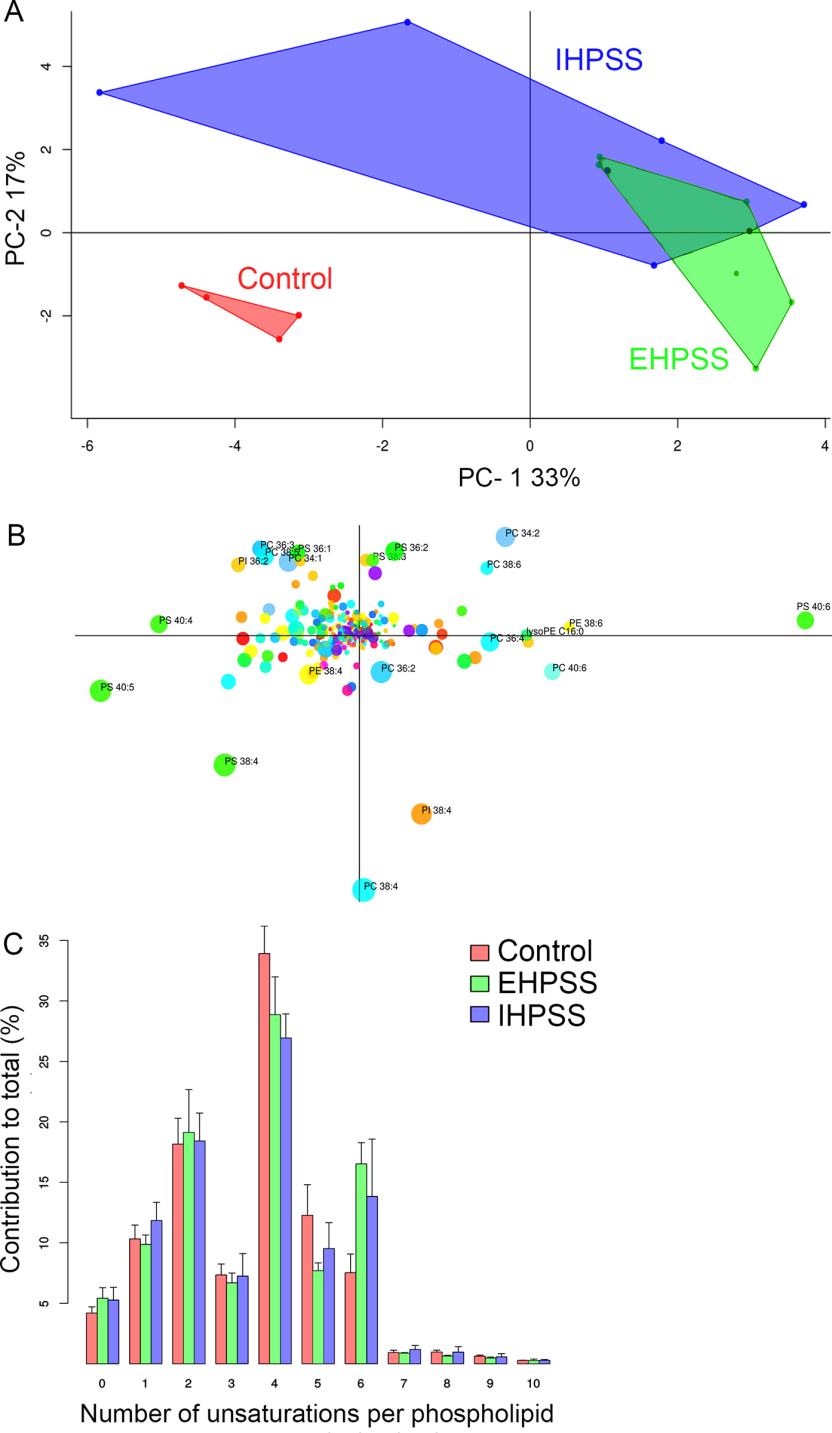
Principal component analysis of phospholipid species. PCA of phospholipid species in hepatic biopsies of healthy (red, *n* = 4), EHPSS (green, *n* = 7), and IHPSS (blue, (*n* = 5) dogs. Resulting scores of the samples (A) using the calculated loadings (B). Lipids are colored according to their lipid class and dot sizes correspond to relative abundance. Degree of unsaturation found in the acyl chains of PL (C). Note the higher levels of acyl chains with four unsaturations in control dogs, at the expense of acyl chains with six unsaturations.

**Fig 4:**
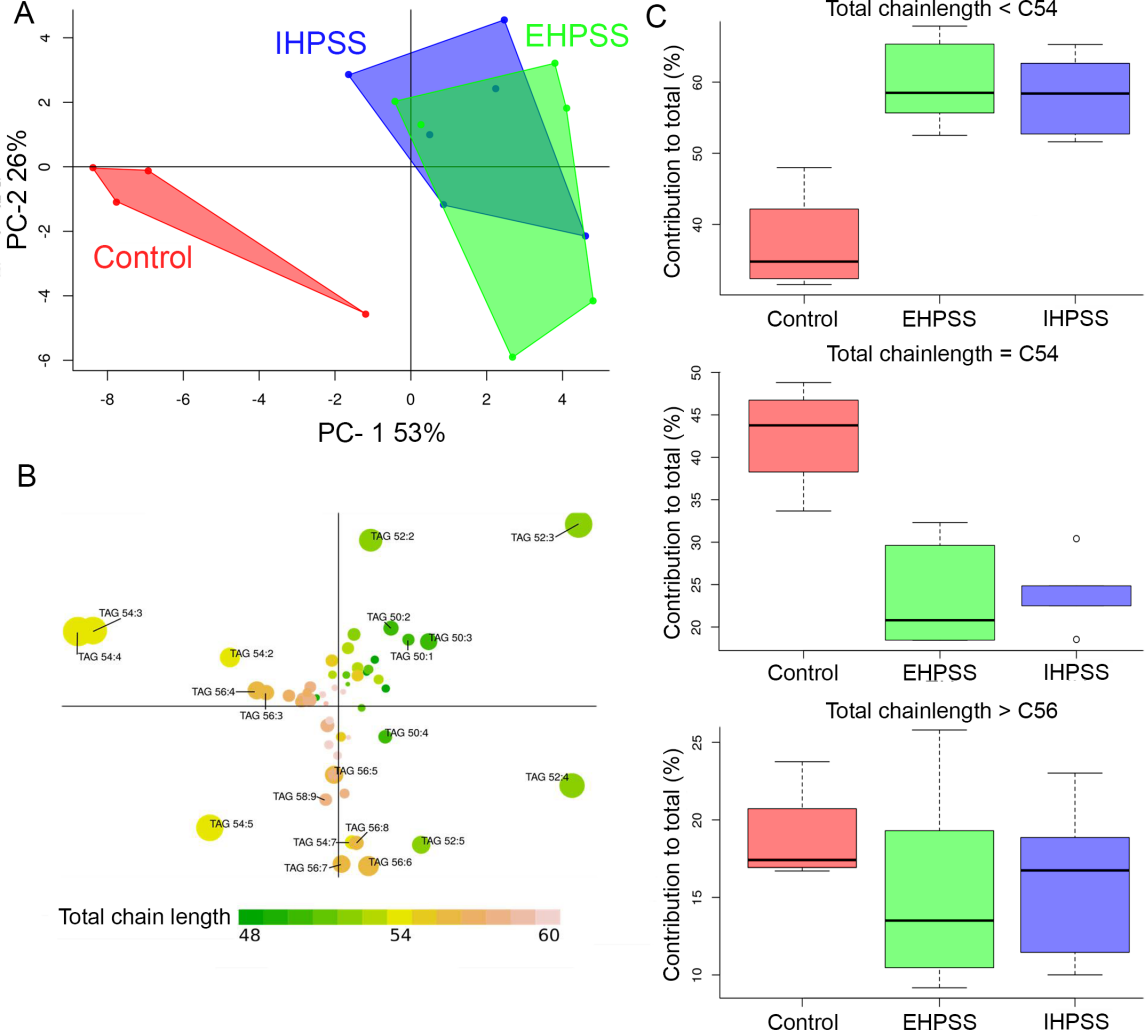
Principal component analysis of neutral lipid species. Representation of the PCA of neutral lipid species in hepatic biopsies of healthy (red, *n* = 4), EHPSS (green, *n* = 7), and IHPSS (blue, *n* = 5) dogs (A). A clear overlap in the TAG part of the lipidome, indicating similarity, is present in EHPSS and IHPSS samples. Calculated loadings of individual TAG species (B) leading to the score plot (A). TAG species are color coded based on the total number of carbon atoms in the acyl chains of TAG species. Note how control dogs have more TAG species with 54 carbon atoms, but less species with shorter acyl chains than dogs with EHPSS or IHPPS (C).

To examine qualitative changes of lipid profiles during the lipid accumulation in liver of shunt dogs, neutral lipids and phospholipids were analysed by HPLC-MS. Examples of such analyses are given in supplementary data (S3 and S4 Figs), respectively. For phospholipids, we observed a clear change in the species profiles of shunt dogs compared to healthy control dogs (Fig 3 and S3 Table). The phospholipidomes of IHPSS and IHPSS were not distinguisable, as can be concluded from the overlap of these samples in the PCA score plot (Fig 3A). When evaluating the phospholipid classes and phospholipid chain length between shunt types or healthy control dogs, no differences are observed (S5 Fig). Interestingly, dogs with a shunt had higher levels of hexaenoic (6 unsaturations per molecule) phospholipid species (Fig 3C) at the expense of tetraenoic (4 double bonds) and, to a lesser extent, pentaenoic (5 double bonds) species compared to the healthy control dogs. From the PCA loading plot (Fig 3B), it can be deduced that this effect is shared among all major phospholipid classes. Arachidonic acid (AA; 20:4, Ω-6) containing species PS 38:4, PC 38:4 and to a lesser extend PE 38:4 and PI 38:4 are located towards the bottom left quadrant of the PCA scores plot (Fig 3B) whereas the docosahexaenoic acid (DHA; 22:6, Ω-3) containing species PS 40:6, PE 38:6 and PC 38:6 are in the top right corner of Fig 3B. The bottom left quadrant (enriched in 20:4, Ω-6) and top right quadrant (enriched in 22:6, Ω-3) correspond to the locations of the control and shunt dogs in the PCA score plots, respectively (Fig 3A). For neutral lipids, a shift towards shorter chain fatty acids (C16:n) is observed in livers of shunt dogs at the expense of more extended fatty acids (C18:n) measured in tissue of healthy dogs (Fig 4 and S4 Table). No difference is observed in TAGs with a chain length of C56 or above between the groups (Fig 4C).

### Similar TAG accumulation in organoids from healthy and shunt livers

**Fig 5:**
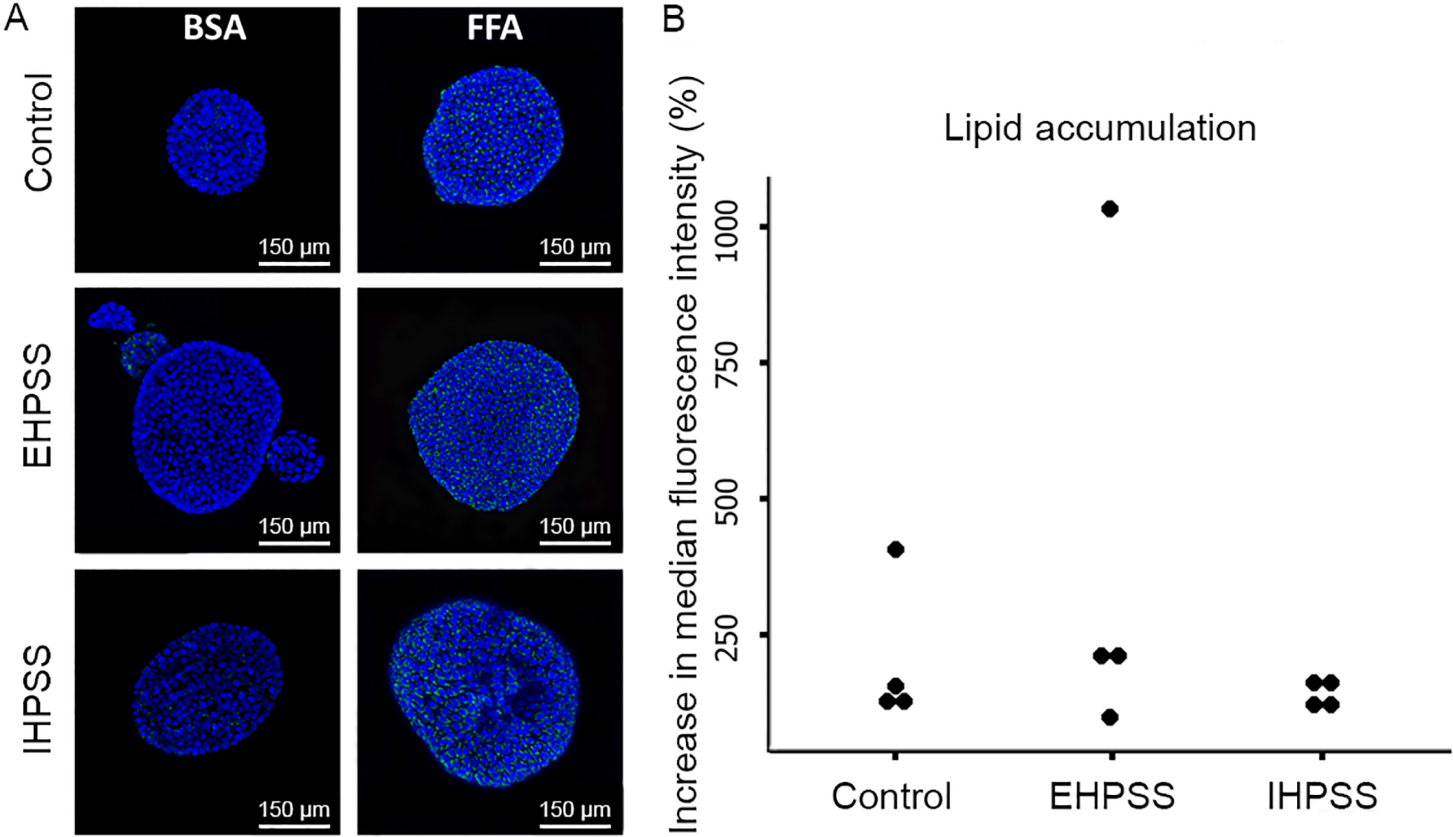
Organoid LD540 fluorescent whole-mount staining. Representative images of LD540 fluorescent whole-mount staining of control (*n* = 4), extrahepatic (EHPSS, *n* = 4) and intrahepatic (IHPSS, *n* = 4) canine organoids cultured in a bovine serum albumin (BSA) medium (control) or the enriched free fatty-acid (FFA) medium for 24 hours (A). Blue represents DAPI–nuclei, green represents LD540 labelled TAGs. The accumulation of LD540 in lipid droplets is more pronounced in the FFA cultured organoids as displayed in a section (A). Quantitative measurement of the total intracellular lipid accumulation was ascertained by flow cytometry analysis (B). Data is expressed as percentage increase in median fluorescent intensity of cells in FFA containing media compared to BSA control (*n* = 4).

Triacylglycerol accumulation in hepatic organoids was evaluated by whole mount LD540 staining. Microscopically, the LD540 accumulation in lipid droplets, displayed in a single section, was more pronounced in the organoids cultured with FFA supplementation and revealed little differences between healthy and both CPSS canine liver organoids regarding basal fatty acid uptake (control medium) or in medium supplemented with FFA (Fig 5A). In order to quantify the total TAG accumulation flow cytometry analysis of whole organoids was performed. The relative increase in median LD540 fluorescence after FFA enrichment was similar in the healthy and shunt groups (Fig 5B) indicating that there were no differences in total TAG accumulation.

## Discussion

This study provides detailed insight into the pathogenesis of hepatic steatosis in CPSS dogs by examination of the quantity of accumulating lipids, mRNA expression of genes involved in lipid metabolism, and lipid profiles in both EHPSS and IHPSS compared to control dogs. To further support our hypothesis, canine organoids of EHPSS, IHPSS and healthy controls demonstrated a similar increase in TAGs after free fatty acids enrichment. Importantly, the results obtained in this study elucidate aspects of steatosis in portosystemic shunting, demonstrate that hepatic steatosis observed in CPSS occurs presumably secondary to portosystemic shunting and finally provide a model to study steatosis in human medicine.

The complex pathogenic mechanisms underlying NAFLD are influenced by multiple factors including genetic, hormonal and nutritional factors [2]. For instance, a missense mutation in human PNPLA3 (patatin-like phospholipase domain-containing 3 protein) is associated with increased hepatic fat deposition and hepatic inflammation, which makes the liver more susceptible for NAFLD [32]. In dogs, hepatic steatosis featured by vacuolar changes within hepatocytes and the presence of lipogranulomas, is a frequently observed finding in liver biopsies of dogs with CPSS [11–13,15]. Once a shunt is attenuated causing restauration of the normal liver blood flow, the lipid accumulation seems to be reversible over time [14]. Only a few descriptive studies have investigated this phenomenon in dogs [15,33,34], but none studied the pathophysiology of steatosis in CPSS.

As extra- and intrahepatic portosystemic shunts have a different genetic background [7], the similarity in the quantity of lipids, gene-expression, and lipid profiles suggests lipid accumulation to be a secondary effect of portosystemic shunting. In addition, the FFA-supplemented organoid cultures did not reveal differences between shunt derived organoids (EHPSS or IHPSS) strengthening the idea of a secondary effect. Age could be an influencing factor on the degree of steatosis in CPSS as the incidence of lipogranulomas (LG) seems to be greater in age-matched dogs with CPSS compared to healthy dogs and LG are generally less observed in dogs under one year of age [33,34]. Another explanation for steatosis occurring secondary to CPSS could be plasma ammonia levels. CPSS are the most frequent cause of hyperammonemia in dogs which, when left untreated, results in hepatic encephalopathy [35]. In case of hyperammonemia, the ammonia is postulated to accumulate in lysosomes. Consequently intralysosomal pH will raise, thereby inhibiting lysosomal enzymes involved in proteolysis and lipid degradation [36,37]. The reduced breakdown of proteins and lipids can contribute to the progressive abnormalities in the brain in case of hepatic encephalopathy [38]. Therefore, the presence of the biomarker ammonia in CPSS [39] might be causative for the secondary lipid accumulations observed in portosystemic shunting. For two reasons it is not likely that insulin resistance, an important factor in the pathogenesis of NAFLD [2], plays a role in steatosis in CPSS dogs. Insulin resistance is associated with hyperinsulinemia [2] and low circulating IGFBP1 levels. Insulin levels vary greatly among diseased dogs [40] and *IGFBP1* levels in this study are induced (Fig 2) rather than reduced. Taken together, the presented data together suggests that steatosis in CPSS dogs is a secondary phenomenon in portosystemic shunting possibly influenced by hyperammonemia.

Gene expression profiling and RT-qPCR validation corroborated the importance of seven lipid related genes to both subtypes of portosystemic shunting. The *FABPI* and *PLIN2* upregulation in extra- and intrahepatic shunts (Fig 2) is probably associated with their lipid related functions and the lipid accumulation in CPSS. *FABP1* serves as a key regulator of hepatic lipid metabolism by enhancing the cellular uptake, transport, and metabolism of fatty-acids [41]. Notably, this protein can bind bile acids which are often increased in CPSS [41]. Increased *PLIN2* expression improves cellular lipid accumulation and regulates (phospho)lipid exchange from lipid droplets [42]. A down-regulation of *HSDB3* was observed, in line with our data, in granulosa cells when FFA concentrations increased [43]. Whether this hepatic down-regulation is directly correlated with FFA content needs to be investigated.

Interestingly, three of the six upregulated lipid related genes (*i*.*e*. *CRP*, *ITIH4*, and *SAA1*), serve as acute phase reactants (APR) which are secreted in response to a variety of acute and chronic inflammatory conditions, in particular regulated by IL-6. CRP is an important nonspecific biochemical marker of inflammation which is synthesized in both liver as well in adipose tissue in the presence of obesity [44]. Besides its role as APR, ITIH4 is associated with hypercholesterolemia [45] and may play an important role in liver regeneration [46]. SAA1 is associated with high-density lipoprotein metabolism, and cholesterol metabolism and transport [47]. This protein is also involved in the formation of amyloid deposits in feline and canine hepatic amyloidosis, triggered by inflammatory conditions [48]. Based on these results, the question raises whether the upregulation of these genes in portosystemic shunting is solely caused by an altered lipid metabolism *per se* or as a consequence of the inflammatory state [49]. The lack of an inflammatory component in hepatic biopsies of CPSS dogs argues in favour of a direct effect of lipid accumulation.

Since the liver plays a vital role in lipid metabolism, any disturbance in the fatty acids and triglycerides pathways leads to an imbalance in the lipid metabolism resulting in hepatic steatosis, and eventually steatohepatitis [50–52]. A plethora of biological effects of omega-3 and -6 fatty acids have been described [53]. Remarkable is the shift towards omega-3 fatty acids at the expense of the omega-6 fatty acids in particular the shift of AA (20:4, Ω-6) to DHA (22:6, Ω-3) in shunt dogs in comparison to healthy control dogs. AA is the main precursor of eicosanoids, which modulate the immune response via a diversity of pathways [54]. Dietary DHA has the capacity to suppress markers of hepatic damage, hepatic inflammation, oxidative stress and fibrosis in LDLR^-/-^ mouse with induced non-alcoholic steatohepatitis [55] and is reported to be beneficial in hepatic encephalopathy [56]. The altered lipid metabolism in elevated levels of DHA in shunt livers compared to healthy control dogs, might therefore be a protective response.

Due to population bottlenecks and inbreeding during the formation of the contemporary dog breeds, canines have a limited phenotypic and genetic diversity which makes the dog population ideal for exploring the genetic basis of a variety of naturally occurring diseases [57–59]. The dog has been proposed as a useful model to study inherited diseases in both canine and human research [60], since they are remarkably similar between canine and human diseases based on phenotypic presentation. This also holds true for CPSS, the same subtypes for intrahepatic and extrahepatic shunts in dogs have been recognized in man, although at a much lower frequency in humans [8,61]. Histological features observed in dogs with CPSS are comparable to human [62] and rats with an induced portacaval shunt [63]. The high prevalence but poorly understood pathogenesis of NAFLD urges the search for reproducible and predictive disease model systems. Therefore, studying lipid loading in CPSS dogs, could in a further stadium serve as a model to study the pathophysiology of steatosis and/or novel treatment modalities preventing lipid accumulation.

In conclusion, this study describes excessive hepatic lipid accumulation in portosystemic shunting. Gene expression profiling indicated that the majority of genes changed in CPSS were involved in lipid metabolism. Different TAG composition and a shift in short chain and omega-3 fatty acids were observed in shunt dogs compared to healthy animals. Despite a different genetic background of extra- and intrahepatic shunts, lipid species observed in both shunt types were almost identical. As cultured organoids derived from healthy and diseased animals accumulate TAGs equally, we suggest that lipid accumulation as observed in shunt livers appears not to be related to primary gene defects in liver shunts, but rather be caused by a secondary effect, possibly ammonia related. Histological features observed in dogs with CPSS are comparable to human [62] and rats with an induced portacaval shunt [63]. Since lipid accumulation is a natural phenomenon in CPSS dogs, these animals might represent a simplified NALFD model.

## Supporting information

S1 Fig Overlap of samples used in different experiments. Identical cohorts indicated in boxed columns have been used in different experiments.
(TIF)

S2 Fig Example of the semi-quantitative scoring system of lipid staining by Oil-red-O. Oil-red-O staining of liver tissue of CPSS and control dogs in the semi-quantitative scoring system graded from low (0) to remarkable high (4). Pictured are examples of mild (1) lipid staining (A), moderate (2) lipid staining (B), severe (3) lipid staining (C), and remarkable high (4) lipid staining (D). The semi-quantitative analysis indicated a significantly higher lipid intensity in liver tissue of dogs with either EHPSS (*P* < 0.01) or IHPSS (*P* < 0.05) compared to healthy dogs.
(TIF)

S3 Fig Example of HPLC-MS analysis of neutral lipids. Base peak chromatogram of the LCMS analysis of neutral lipids, showing the partial separation of TAG molecular species (A). Coeluting TAG species can be identified in the MS spectrum (B). The spectrum in the bottom panel was recorded at the timepoint indicated by an arrow in the top panel. The m/z signals correspond to TAG species as listed in S4 Table.
(TIF)

S4 Fig Example of HPLC-MS analysis of phospholipids. Base peak chromatogram recorded during the separation of phospholipid classes by hydrophilic interaction liquid chromatography (HILIC) (A). Lipid species contributing to a lipid class can be inferred from the mass spectrum recorded during elution as illustrated for PI (B). Total phospholipid profiles are listed in “S3 Table”.
(TIF)

S5 Fig **Phospholipid species (A) and total carbon length of the acyl chains (B).** In phospholipid analysis no differences in chain length or classes between shunt types or healthy control dogs are observed. BMP, bis-monoacylglycerol phosphate; lysoPC, lysophosphatidylcholine; lysoPE, lysophosphatidylethanolamine; PC, Phosphatidylcholine; PE, Phosphatidylethanolamine; PG, Phosphatidylglycerol; SM, Sphingomyelin.
(TIF)

S1 Table Primers used for quantitative real-time PCR (RT-qPCR). *CRP*, *C-reactive protein; ELOVL5*, *ELOVL fatty acid elongase 5; ELOVL6*, *ELOVL fatty acid elongase 6; FABP1*, *Fatty acid binding protein 1; HSD3B*, *Hydroxy-delta-5-steroid dehydrogenase 3-beta; IGFBP1*, *Insulin-like growth factor binding protein 1; ITIH3*, *Inter-alpha-trypsin inhibitor heavy chain 3; ITIH4*, *Inter-alpha-trypsin inhibitor heavy chain 4; PLIN2*, *Perilipin 2; SAA1*, *Serum amyloid A1; SEC14L3*, *SEC14-like lipid binding 3; GAPDH*, *Glyceraldehyde-3-phosphatedehydrogenase; HNRPH*, *Heterogeneous nuclear ribonucleoprotein H; RPS19*, *Ribosomal protein S19; RPS5*, *Ribosomal protein S5*.
(PDF)

S2 Table Gene list. Top 24 list of most up and down regulated genes by gene-expression profiling of hepatic tissue of dogs with a congenital portosystemic shunt compared to healthy liver samples. Fold change of the microarray (MA) and quantitative reversed transcriptase PCR (RT-qPCR) are displayed.
(PDF)

S3 Table Contribution of individual phospholipid species to the LCMS analysis of the phospholipidome. (PDF)

S4 Table Contribution of individual TAG lipid species to the total [M+H]+ signal of TAG in LCMS analysis with atmospheric pressure chemical ionization (APCI). Only TAG species contributing more than 0.015% on average are included.
(PDF)

## References

[1] Rinella ME. Nonalcoholic fatty liver disease: a systematic review. JAMA 2015 6 9;313(22):2263–2273. doi: 10.1001/jama.2015.5370 26057287 10.1001/jama.2015.5370

[2] Carr RM, Oranu A, Khungar V. Nonalcoholic Fatty Liver Disease: Pathophysiology and Management. Gastroenterol Clin North Am 2016 12;45(4):639–652. doi: 10.1016/j.gtc.2016.07.003 27837778 10.1016/j.gtc.2016.07.003 PMC5127277

[3] Mikolasevic I, Milic S, Turk Wensveen T, Grgic I, Jakopcic I, Stimac D, et al Nonalcoholic fatty liver disease—A multisystem disease? World J Gastroenterol 2016 11 21;22(43):9488–9505. doi: 10.3748/wjg.v22.i43.9488 27920470 10.3748/wjg.v22.i43.9488 PMC5116593

[4] Valenti L, Bugianesi E, Pajvani U, Targher G. Nonalcoholic fatty liver disease: cause or consequence of type 2 diabetes? Liver Int 2016 11;36(11):1563–1579. doi: 10.1111/liv.13185 27276701 10.1111/liv.13185

[5] Mann JP, Semple RK, Armstrong MJ. How Useful Are Monogenic Rodent Models for the Study of Human Non-Alcoholic Fatty Liver Disease? Front Endocrinol (Lausanne) 2016 11 16;7:145. 27899914 10.3389/fendo.2016.00145 PMC5110950

[6] Stringer MD. The Clinical Anatomy of Congenital Portosystemic Venous Shunts. Clinical Anatomy 2008;21:147–157. doi: 10.1002/ca.20574 18161055 10.1002/ca.20574

[7] Van den Bossche L, van Steenbeek FG. Canine congenital portosystemic shunts: Disconnections dissected. Vet J 2016 5;211:14–20. doi: 10.1016/j.tvjl.2015.09.025 27061656 10.1016/j.tvjl.2015.09.025

[8] van Steenbeek FG, van den Bossche L, Leegwater PA, Rothuizen J. Inherited liver shunts in dogs elucidate pathways regulating embryonic development and clinical disorders of the portal vein. Mamm Genome 2012 2;23(1–2):76–84. doi: 10.1007/s00335-011-9364-0 22052005 10.1007/s00335-011-9364-0 PMC3275728

[9] van den Ingh TS, Rothuizen J, Meyer HP. Circulatory disorders of the liver in dogs and cats. Vet Q 1995 6;17(2):70–76. doi: 10.1080/01652176.1995.9694536 7571284 10.1080/01652176.1995.9694536

[10] Winkler JT, Bohling MW, Tillson DM, Wright JC, Ballagas AJ. Portosystemic shunts: diagnosis, prognosis, and treatment of 64 cases (1993–2001). J Am Anim Hosp Assoc 2003 Mar-Apr;39(2):169–185. doi: 10.5326/0390169 12617545 10.5326/0390169

[11] Cullen JM, van den Ingh TSGAM, Bunch SE, Rothuizen J, Washabau RJ, Desmet VJ. Chapter 4—Morphological classification of circulatory disorders of the canine and feline liver WSAVA Standards for Clinical and Histological Diagnosis of Canine and Feline Liver Diseases: Elsevier; 2006 p. 41–59.

[12] Baade S, Aupperle H, Grevel V, Schoon H-. Histopathological and Immunohistochemical Investigations of Hepatic Lesions Associated with Congenital Portosystemic Shunt in Dogs. J Comp Path 2006;134:80–90. doi: 10.1016/j.jcpa.2005.07.003 16423574 10.1016/j.jcpa.2005.07.003

[13] Parker JS, Monnet E, Powers BE, Twedt DC. Histologic examination of hepatic biopsy samples as a prognostic indicator in dogs undergoing surgical correction of congenital portosystemic shunts: 64 cases (1997–2005). Journal of the American Veterinary Medical Association 2008;232(10):1511–1514. doi: 10.2460/javma.232.10.1511 18479241 10.2460/javma.232.10.1511

[14] Lee KC, Winstanley A, House JV, Lipscomb V, Lamb C, Gregory S, et al Association between hepatic histopathologic lesions and clinical findings in dogs undergoing surgical attenuation of a congenital portosystemic shunt: 38 cases (2000–2004). J Am Vet Med Assoc 2011 9 1;239(5):638–645. doi: 10.2460/javma.239.5.638 21879964 10.2460/javma.239.5.638

[15] Hunt GB, Luff JA, Daniel L, Van den Bergh R. Evaluation of hepatic steatosis in dogs with congenital portosystemic shunts using Oil Red O staining. Vet Pathol 2013 11;50(6):1109–1115. doi: 10.1177/0300985813481609 23528942 10.1177/0300985813481609 PMC4445129

[16] van Steenbeek FG, Leegwater PA, van Sluijs FJ, Heuven HC, Rothuizen J. Evidence of inheritance of intrahepatic portosystemic shunts in Irish Wolfhounds. J Vet Intern Med 2009 Jul-Aug;23(4):950–952. doi: 10.1111/j.1939-1676.2009.0319.x 19496918 10.1111/j.1939-1676.2009.0319.x

[17] van Straten G, Spee B, Rothuizen J, van Straten M, Favier RP. Diagnostic value of the rectal ammonia tolerance test, fasting plasma ammonia and fasting plasma bile acids for canine portosystemic shunting. Vet J 2015 6;204(3):282–286. doi: 10.1016/j.tvjl.2015.04.020 25959128 10.1016/j.tvjl.2015.04.020

[18] Wolschrijn CF, Mahapokai W, Rothuizen J, Meyer HP, van Sluijs FJ. Gauged attenuation of congenital portosystemic shunts: results in 160 dogs and 15 cats. Vet Q 2000 4;22(2):94–98. doi: 10.1080/01652176.2000.9695032 10789517 10.1080/01652176.2000.9695032

[19] Huisman A, Looijen A, van den Brink SM, van Diest PJ. Creation of a fully digital pathology slide archive by high-volume tissue slide scanning. Hum Pathol 2010 May;41(5):751–757. doi: 10.1016/j.humpath.2009.08.026 20129646 10.1016/j.humpath.2009.08.026

[20] van Steenbeek FG, Van den Bossche L, Grinwis GC, Kummeling A, van Gils IH, Koerkamp MJ, et al Aberrant gene expression in dogs with portosystemic shunts. PLoS One 2013;8(2):e57662 doi: 10.1371/journal.pone.0057662 23451256 10.1371/journal.pone.0057662 PMC3581512

[21] Bustin SA, Beaulieu JF, Huggett J, Jaggi R, Kibenge FS, Olsvik PA, et al MIQE precis: Practical implementation of minimum standard guidelines for fluorescence-based quantitative real-time PCR experiments. BMC Mol Biol 2010 9 21;11:74-2199-11-74. 10.1186/1471-2199-11-74 PMC2955025 20858237

[22] Bligh EG, Dyer WJ. A rapid method of total lipid extraction and purification. Can J Biochem Physiol 1959 8;37(8):911–917. doi: 10.1139/o59-099 13671378 10.1139/o59-099

[23] Retra K, Bleijerveld OB, van Gestel RA, Tielens AG, van Hellemond JJ, Brouwers JF. A simple and universal method for the separation and identification of phospholipid molecular species. Rapid Commun Mass Spectrom 2008 6;22(12):1853–1862. doi: 10.1002/rcm.3562 18470873 10.1002/rcm.3562

[24] Jeucken A, Brouwers JF. Liquid Chromatography- Mass Spectrometry of Glycerophospholipids In: Wenk MR, editor. Encyclopedia of Lipidomics: Springer; in press.

[25] Nantasanti S, Spee B, Kruitwagen HS, Chen C, Geijsen N, Oosterhoff LA, et al Disease Modeling and Gene Therapy of Copper Storage Disease in Canine Hepatic Organoids. Stem Cell Reports 2015 11 10;5(5):895–907. doi: 10.1016/j.stemcr.2015.09.002 26455412 10.1016/j.stemcr.2015.09.002 PMC4649105

[26] Willert K, Brown JD, Danenberg E, Duncan AW, Weissman IL, Reya T, et al Wnt proteins are lipid-modified and can act as stem cell growth factors. Nature 2003 5 22;423(6938):448–452. doi: 10.1038/nature01611 12717451 10.1038/nature01611

[27] Spandl J, White DJ, Peychl J, Thiele C. Live cell multicolor imaging of lipid droplets with a new dye, LD540. Traffic 2009 11;10(11):1579–1584. doi: 10.1111/j.1600-0854.2009.00980.x 19765264 10.1111/j.1600-0854.2009.00980.x

[28] Wu H, Kerr MK, Cui X, Churchill GA. MAANOVA: A Software Package for the Analysis of Spotted cDNA Microarray Experiments In: parmigiani GG, Garret ES, Irizarri RA, Zeger SL, editors. The Analysis of Gene Expression Data; methods and software New York: Springer; 2003 p. 313–339.

[29] Smith CA, Want EJ, O'Maille G, Abagyan R, Siuzdak G. XCMS: processing mass spectrometry data for metabolite profiling using nonlinear peak alignment, matching, and identification. Anal Chem 2006 2 1;78(3):779–787. doi: 10.1021/ac051437y 16448051 10.1021/ac051437y

[30] R Core Team. R: A language and environment for statistical computing R Foundation for Statistical Computing, Vienna, Austria 2016; Available at: https://www.R-project.org/.

[31] Stacklies W, Redestig H, Scholz M, Walther D, Selbig J. pcaMethods—a bioconductor package providing PCA methods for incomplete data. Bioinformatics 2007 5 1;23(9):1164–1167. doi: 10.1093/bioinformatics/btm069 17344241 10.1093/bioinformatics/btm069

[32] Romeo S, Kozlitina J, Xing C, Pertsemlidis A, Cox D, Pennacchio LA, et al Genetic variation in PNPLA3 confers susceptibility to nonalcoholic fatty liver disease. Nat Genet 2008 12;40(12):1461–1465. doi: 10.1038/ng.257 18820647 10.1038/ng.257 PMC2597056

[33] Isobe K, Matsunaga S, Nakayama H, Uetsuka K. Histopathological characteristics of hepatic lipogranulomas with portosystemic shunt in dogs. J Vet Med Sci 2008 2;70(2):133–138. 18319572 10.1292/jvms.70.133

[34] Hunt GB, Luff J, Daniel L, Zwingenberger A. Does hepatic steatosis have an impact on the short term hepatic response after complete attenuation of congenital extrahepatic portosystemic shunts? A prospective study of 20 dogs. Vet Surg 2014 11;43(8):920–925. doi: 10.1111/j.1532-950X.2014.12197.x 24819233 10.1111/j.1532-950X.2014.12197.x

[35] van Straten G, van Steenbeek FG, Grinwis GC, Favier RP, Kummeling A, van Gils IH, et al Aberrant expression and distribution of enzymes of the urea cycle and other ammonia metabolizing pathways in dogs with congenital portosystemic shunts. PLoS One 2014 6 19;9(6):e100077 doi: 10.1371/journal.pone.0100077 24945279 10.1371/journal.pone.0100077 PMC4063766

[36] Lüllmann-Rauch R. Drug-induced lysosomal storage disorders In: Dingle JT, Jacques PJ, Shaw IH, editors. Lysomes in Applied Biology and Therapeutics Amsterdam, Netherlands: North-Holland Publishing Company; 1979 p. 49–130.

[37] Seglen PO. Inhibitors of lysosomal function. Methods Enzymol 1983;96:737–764. 6361463 10.1016/s0076-6879(83)96063-9

[38] Dienel GA, Cruz NF. Reduced clearance of proteins labeled with diisopropylfluorophosphate in portacaval-shunted rats. Metab Brain Dis 2014 12;29(4):1041–1052. doi: 10.1007/s11011-013-9442-y 24154686 10.1007/s11011-013-9442-y PMC4000281

[39] Kerr MG, van Doorn T. Mass screening of Irish wolfhound puppies for portosystemic shunts by the dynamic bile acid test. Vet Rec 1999 6 19;144(25):693–696. 10420483 10.1136/vr.144.25.693

[40] Collings AJ, Gow AG, Marques A, Yool D, Furneaux R, Mellanby R, et al A prospective study of basal insulin concentrations in dogs with congenital portosystemic shunts. J Small Anim Pract 2012 4;53(4):228–233. doi: 10.1111/j.1748-5827.2011.01192.x 22417097 10.1111/j.1748-5827.2011.01192.x

[41] Hertzel AV, Bernlohr DA. The mammalian fatty acid-binding protein multigene family: molecular and genetic insights into function. Trends Endocrinol Metab 2000 7;11(5):175–180. 10856918 10.1016/s1043-2760(00)00257-5

[42] McIntosh AL, Senthivinayagam S, Moon KC, Gupta S, Lwande JS, Murphy CC, et al Direct interaction of Plin2 with lipids on the surface of lipid droplets: a live cell FRET analysis. Am J Physiol Cell Physiol 2012 10 1;303(7):C728–42. doi: 10.1152/ajpcell.00448.2011 22744009 10.1152/ajpcell.00448.2011 PMC3469596

[43] Yenuganti VR, Viergutz T, Vanselow J. Oleic acid induces specific alterations in the morphology, gene expression and steroid hormone production of cultured bovine granulosa cells. Gen Comp Endocrinol 2016 6 1;232:134–144. doi: 10.1016/j.ygcen.2016.04.020 27118706 10.1016/j.ygcen.2016.04.020

[44] Anty R, Bekri S, Luciani N, Saint-Paul MC, Dahman M, Iannelli A, et al The inflammatory C-reactive protein is increased in both liver and adipose tissue in severely obese patients independently from metabolic syndrome, Type 2 diabetes, and NASH. Am J Gastroenterol 2006 8;101(8):1824–1833. doi: 10.1111/j.1572-0241.2006.00724.x 16790033 10.1111/j.1572-0241.2006.00724.x

[45] Fujita Y, Ezura Y, Emi M, Sato K, Takada D, Iino Y, et al Hypercholesterolemia associated with splice-junction variation of inter-alpha-trypsin inhibitor heavy chain 4 (ITIH4) gene. J Hum Genet 2004;49(1):24–28. doi: 10.1007/s10038-003-0101-8 14661079 10.1007/s10038-003-0101-8

[46] Bhanumathy CD, Tang Y, Monga SP, Katuri V, Cox JA, Mishra B, et al Itih-4, a serine protease inhibitor regulated in interleukin-6-dependent liver formation: role in liver development and regeneration. Dev Dyn 2002 1;223(1):59–69. doi: 10.1002/dvdy.1235 11803570 10.1002/dvdy.1235

[47] Urieli-Shoval S, Linke RP, Matzner Y. Expression and function of serum amyloid A, a major acute-phase protein, in normal and disease states. Curr Opin Hematol 2000 1;7(1):64–69. 10608507 10.1097/00062752-200001000-00012

[48] Ceron JJ, Eckersall PD, Martynez-Subiela S. Acute phase proteins in dogs and cats: current knowledge and future perspectives. Vet Clin Pathol 2005 6;34(2):85–99. 15902658 10.1111/j.1939-165x.2005.tb00019.x

[49] Boden G. Fatty acid-induced inflammation and insulin resistance in skeletal muscle and liver. Curr Diab Rep 2006 6;6(3):177–181. 16898568 10.1007/s11892-006-0031-x

[50] Koteish A, Mae Diehl A. Animal models of steatohepatitis. Best Pract Res Clin Gastroenterol 2002 10;16(5):679–690. 12406439 10.1053/bega.2002.0332

[51] Stalker MJ, Hayes MAT. Jubb, Kennedy and Palmer’s Pathology of Domestic Animals; Liver and biliary system In: Grant Maxie M, editor. Jubb, Kennedy and Palmer’s Pathology of Domestic Animals. 5th edition ed.: Saunders Elsevier; 2007 p. 297–315.

[52] Postic C, Girard J. The role of the lipogenic pathway in the development of hepatic steatosis. Diabetes Metab 2008 12;34(6 Pt 2):643–648. 19195625 10.1016/S1262-3636(08)74599-3

[53] Schmitz G, Ecker J. The opposing effects of n-3 and n-6 fatty acids. Prog Lipid Res 2008 3;47(2):147–155. doi: 10.1016/j.plipres.2007.12.004 18198131 10.1016/j.plipres.2007.12.004

[54] Harizi H, Corcuff JB, Gualde N. Arachidonic-acid-derived eicosanoids: roles in biology and immunopathology. Trends Mol Med 2008 10;14(10):461–469. doi: 10.1016/j.molmed.2008.08.005 18774339 10.1016/j.molmed.2008.08.005

[55] Depner CM, Philbrick KA, Jump DB. Docosahexaenoic acid attenuates hepatic inflammation, oxidative stress, and fibrosis without decreasing hepatosteatosis in a Ldlr(-/-) mouse model of western diet-induced nonalcoholic steatohepatitis. J Nutr 2013 3;143(3):315–323. doi: 10.3945/jn.112.171322 23303872 10.3945/jn.112.171322 PMC3713021

[56] Staziaki PV, Marques CM, Delattre AM, de Paula Cioni B, Rufino M, Dos Santos FV, et al Fish oil has beneficial effects on behavior impairment and oxidative stress in rats subjected to a hepatic encephalopathy model. CNS Neurol Disord Drug Targets 2013 2 1;12(1):84–93. 23244424 10.2174/1871527311312010014

[57] Ostrander EA, Kruglyak L. Unleashing the canine genome. Genome Res 2000 9;10(9):1271–1274. 10984444 10.1101/gr.155900

[58] Parker HG, Kim LV, Sutter NB, Carlson S, Lorentzen TD, Malek TB, et al Genetic structure of the purebred domestic dog. Science 2004 5 21;304(5674):1160–1164. doi: 10.1126/science.1097406 15155949 10.1126/science.1097406

[59] Parker HG, Ostrander EA. Canine genomics and genetics: running with the pack. PLoS Genet 2005 11;1(5):e58 doi: 10.1371/journal.pgen.0010058 16311623 10.1371/journal.pgen.0010058 PMC1287952

[60] van Steenbeek FG, Hytonen MK, Leegwater PA, Lohi H. The canine era: the rise of a biomedical model. Anim Genet 2016 10;47(5):519–527. doi: 10.1111/age.12460 27324307 10.1111/age.12460

[61] Sokollik C, Bandsma RH, Gana JC, van den Heuvel M, Ling SC. Congenital portosystemic shunt: characterization of a multisystem disease. J Pediatr Gastroenterol Nutr 2013 6;56(6):675–681. doi: 10.1097/MPG.0b013e31828b3750 23412540 10.1097/MPG.0b013e31828b3750

[62] Lisovsky M, Konstas AA, Misdraji J. Congenital extrahepatic portosystemic shunts (Abernethy malformation): a histopathologic evaluation. Am J Surg Pathol 2011 9;35(9):1381–1390. doi: 10.1097/PAS.0b013e3182230ce4 21836489 10.1097/PAS.0b013e3182230ce4

[63] Aller MA, Martinez V, Corcuera MT, Benito J, Traver E, Gomez-Aguado F, et al Liver impairment after portacaval shunt in the rat: the loss of protective role of mast cells? Acta Histochem 2012 7;114(4):301–310. doi: 10.1016/j.acthis.2011.06.011 21937094 10.1016/j.acthis.2011.06.011

