# Supplementary figures and images for: Aberrant hepatic lipid storage and metabolism in canine portosystemic shunts
Source: PLoS One. 2017 Oct 19;12(10):e0186491. doi: 10.1371/journal.pone.0186491 (PMC5648188; doi:10.1371/journal.pone.0186491)

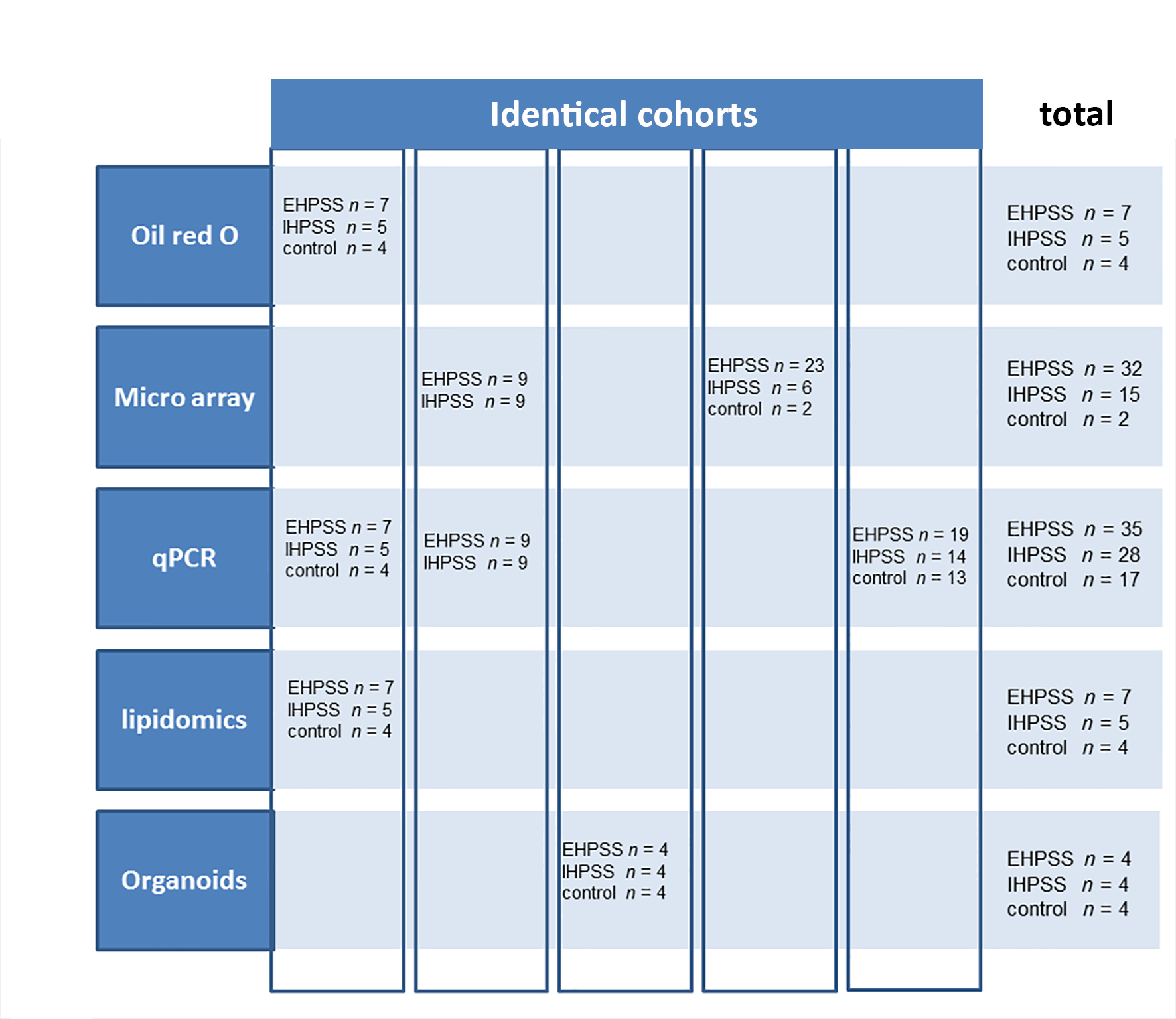

Supplement: S1 Fig — Identical cohorts indicated in boxed columns have been used in different experiments. (TIF) [file pone.0186491.s001.tif]

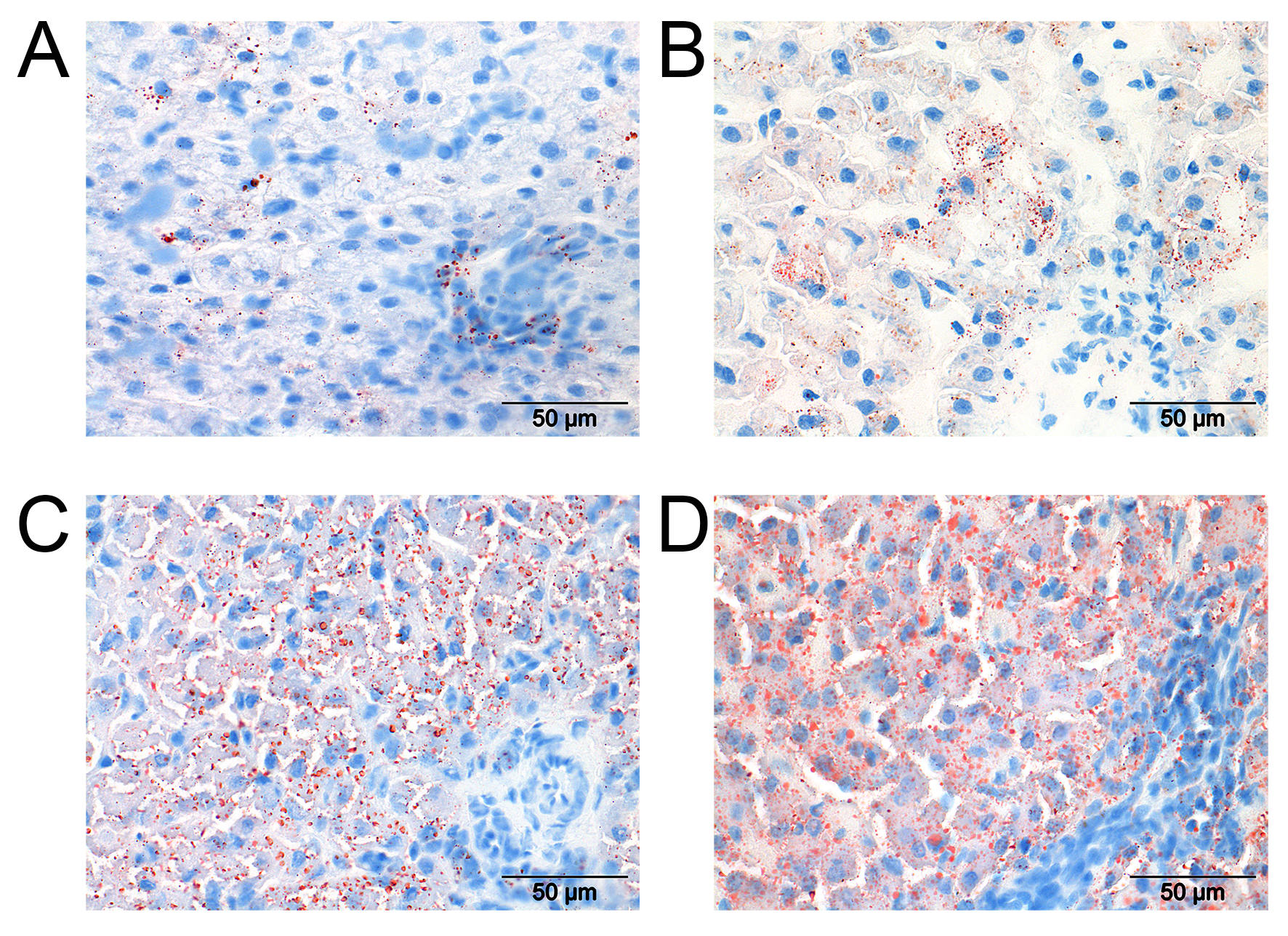

Supplement: S2 Fig — Oil-red-O staining of liver tissue of CPSS and control dogs in the semi-quantitative scoring system graded from low (0) to remarkable high (4). Pictured are examples of mild (1) lipid staining (A), moderate (2) lipid staining (B), severe (3) lipid staining (C), and remarkable high (4) lipid staining (D). The semi-quantitative analysis indicated a significantly higher lipid intensity in liver tissue of dogs with either EHPSS (P < 0.01) or IHPSS (P < 0.05) compared to healthy dogs. (TIF) [file pone.0186491.s002.tif]

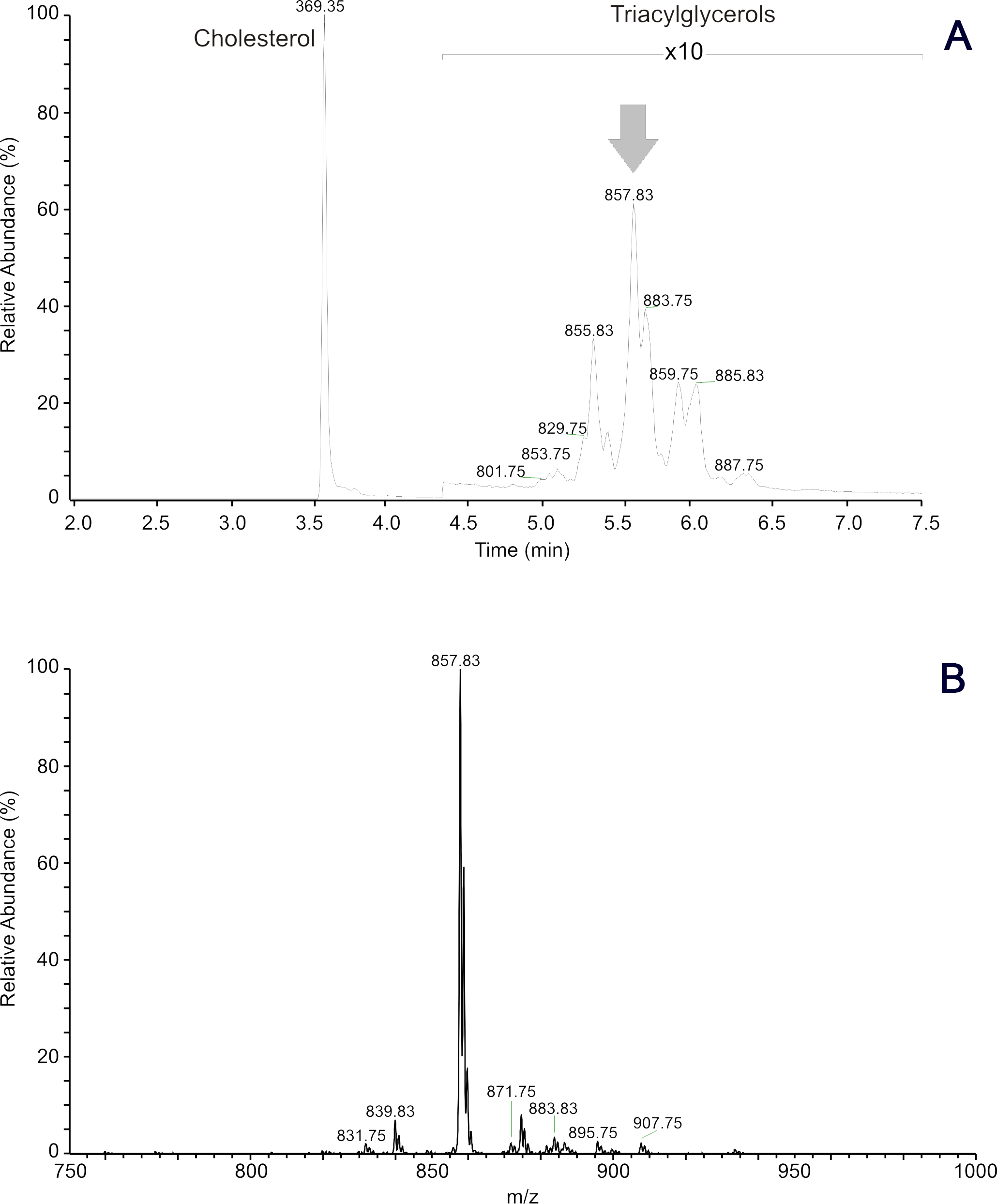

Supplement: S3 Fig — Base peak chromatogram of the LCMS analysis of neutral lipids, showing the partial separation of TAG molecular species (A). Coeluting TAG species can be identified in the MS spectrum (B). The spectrum in the bottom panel was recorded at the timepoint indicated by an arrow in the top panel. The m/z signals correspond to TAG species as listed in S4 Table. (TIF) [file pone.0186491.s003.tif]

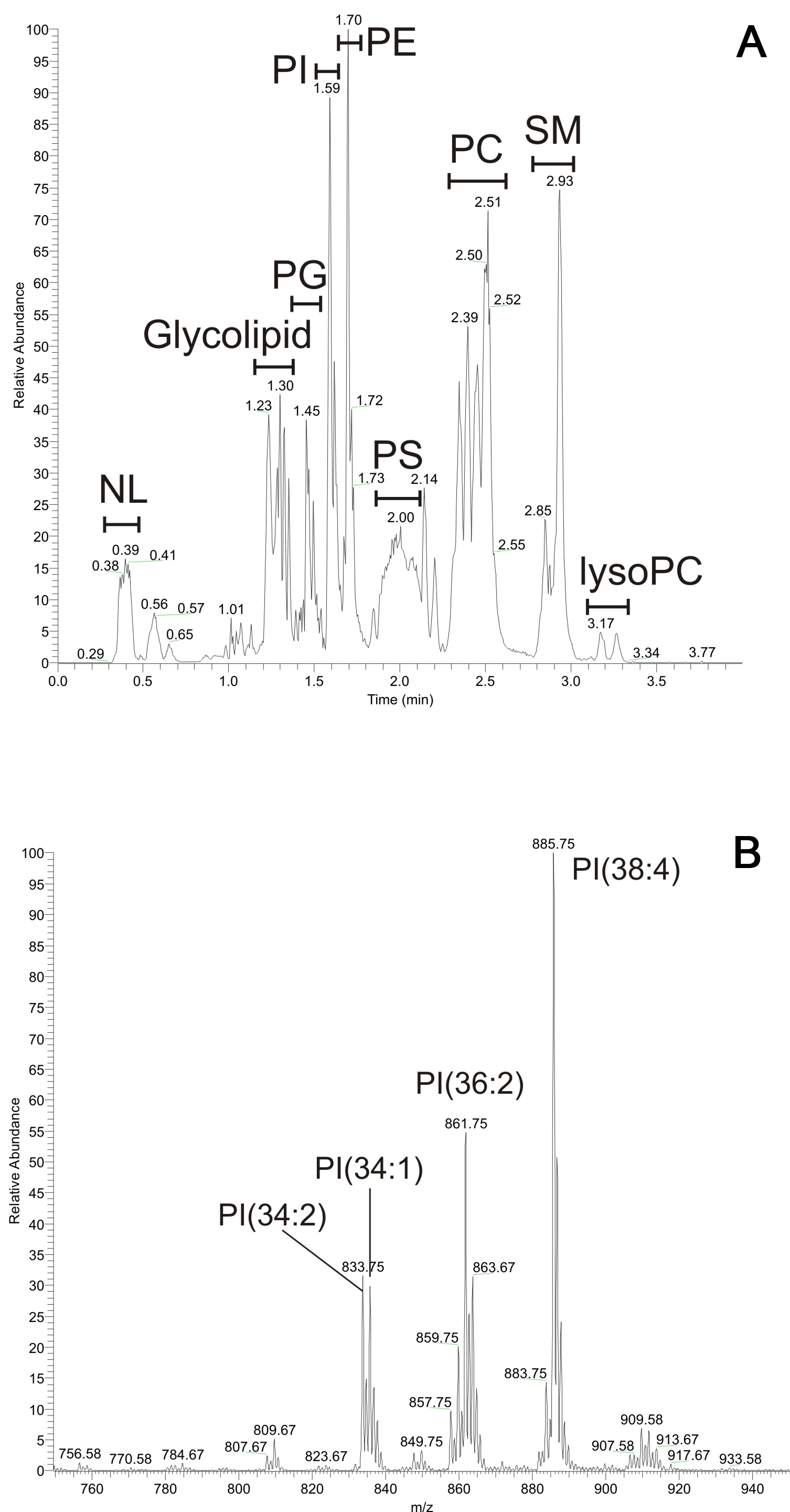

Supplement: S4 Fig — Base peak chromatogram recorded during the separation of phospholipid classes by hydrophilic interaction liquid chromatography (HILIC) (A). Lipid species contributing to a lipid class can be inferred from the mass spectrum recorded during elution as illustrated for PI (B). Total phospholipid profiles are listed in “S3 Table”. (TIF) [file pone.0186491.s004.tif]

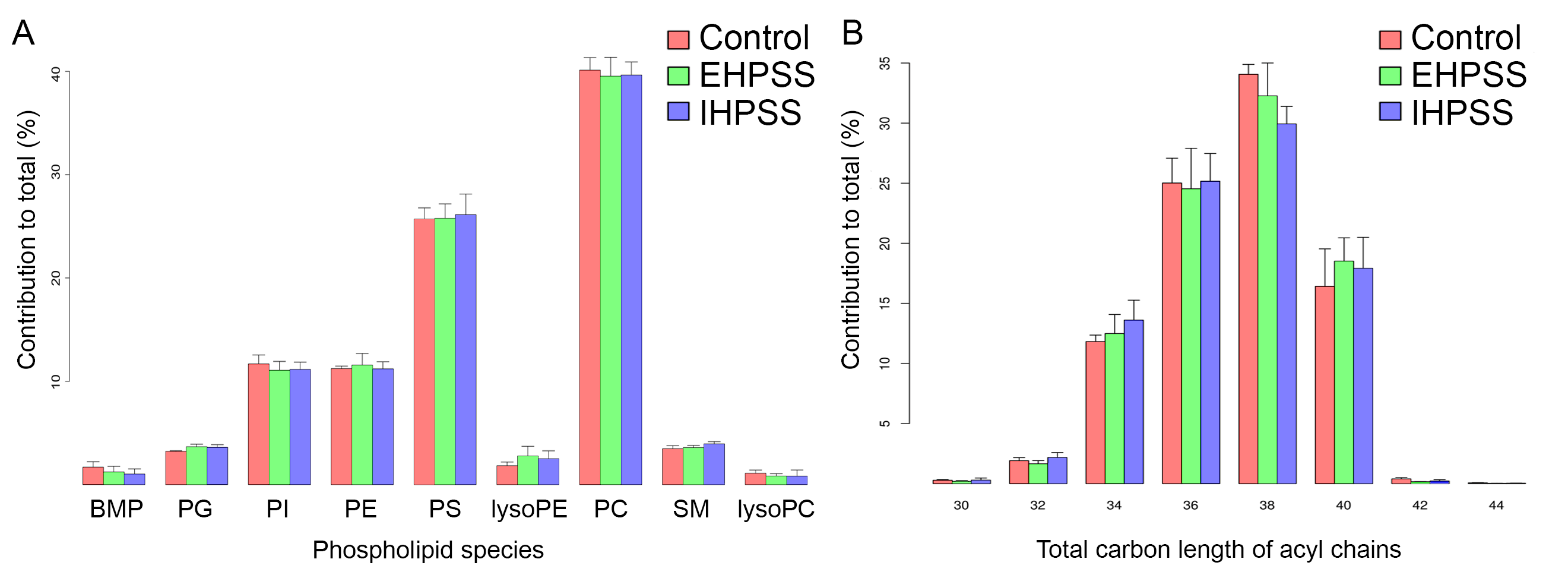

Supplement: S5 Fig — Phospholipid species (A) and total carbon length of the acyl chains (B). In phospholipid analysis no differences in chain length or classes between shunt types or healthy control dogs are observed. BMP, bis-monoacylglycerol phosphate; lysoPC, lysophosphatidylcholine; lysoPE, lysophosphatidylethanolamine; PC, Phosphatidylcholine; PE, Phosphatidylethanolamine; PG, Phosphatidylglycerol; SM, Sphingomyelin. (TIF) [file pone.0186491.s005.tif]
