# Supplementary material for: Aberrant hepatic lipid storage and metabolism in canine portosystemic shunts
Source: PLoS One. 2017 Oct 19;12(10):e0186491. doi: 10.1371/journal.pone.0186491 (PMC5648188; doi:10.1371/journal.pone.0186491)
Supplement: S1 Table — CRP, C-reactive protein; ELOVL5, ELOVL fatty acid elongase 5; ELOVL6, ELOVL fatty acid elongase 6; FABP1, Fatty acid binding protein 1; HSD3B, Hydroxy-delta-5-steroid dehydrogenase 3-beta; IGFBP1, Insulin-like growth factor binding protein 1; ITIH3, Inter-alpha-trypsin inhibitor heavy chain 3; ITIH4, Inter-alpha-trypsin inhibitor heavy chain 4; PLIN2, Perilipin 2; SAA1, Serum amyloid A1; SEC14L3, SEC14-like lipid binding 3; GAPDH, Glyceraldehyde-3-phosphatedehydrogenase; HNRPH, Heterogeneous nuclear ribonucleoprotein H; RPS19, Ribosomal protein S19; RPS5, Ribosomal protein S5. (PDF) [file pone.0186491.s006.pdf]

Gene	Ensemble Transcript ID	F/R	Sequence	Tm (°C)	Amplicon size (bp)
CRP	ENSCAFT00000018706	F	5'-GGTCCTCATGAACCTCC-3'	65	161
		R	5'-GTCAAGTCCGTATAGACCT-3'		
ELOVL5	ENSCAFT00000044079	F	5'-CTGTGAGTTAGTGACGGGA-3'	65	110
		R	5'-TAGTACCACCAGAGGACAC-3'		
ELOVL6	ENSCAFT00000018328	F	5'-CAAAGCACCCGAACTAGGA-3'	59	101
		R	5'-CAGGAGTACAGGAGCACAG-3'		
FABP1	ENSCAFT00000011880	F	5'-GTTCCAAAGTGATCCAGAATGAG-3'	63	107
		R	5'-GCTTATTGTCACCTTCCATCTG-3'		
HSD3B	ENSCAFT00000015969	F	5'-CAGTTGTCATTCACACCG-3'	62	87
		R	5'-TGAGTACCCTTCAGATTGAC-3'		
IGFBP1	ENSCAFT00000019512	F	5'-AGATCCGACGACTCCGA-3'	60.5	196
		R	5'-TACACGCACCAGCAGAG-3'		
ITIH3	ENSCAFT00000023969	F	5'-CTTCATCATCCAAGTTCCGCA-3	62	149
		R	5'-GAGTTAGAGTCGCCTCCCT-3'		
ITIH4	ENSCAFT00000039621	F	5'-CGAATGCCCTCACCATCTC-3'	59	106
		R	5'-GACAATGAAGTTGCCATCCAG-3'		
PLIN2	ENSCAFT00000002516	F	5'-AATGCACTCACCAAATCAG-3'	64	105
		R	5'-TCTGAACTGTATCAAACCCT-3'		
SAA1	ENSCAFT00000014555	F	5'-TTTCTGTTCCTTGGTCCTG-3'	62	141
		R	5'-GGCATGGAAGTATTTGTCTG-3'		
SEC14L3	ENSCAFT00000020256	F	5'-AAGTCCATGTATGTGCGG-3'	63	131
		R	5'-AGATGAGAACTGCCACCT-3'		
GAPDH	ENSCAFT00000037560	F	5′-TGTCCCCACCCCCAATGTATC-3′	58	100
		R	5′-CTCCGATGCCTGCTTCACTACCTT-3′		
HNRPH	ENSCAFT00000028063	F	5′-CTCACTATGATCCACCACG-3′	61	151
		R	5′-TAGCCTCCATAACCTCCAC-3′		
RPS19	ENSCAFT00000008009	F	5′-CCTTCCTCAAAAAGTCTGGG-3′	61	95
		R	5′-GTTCTCATCGTAGGGAGCAAG-3′		
RPS5	ENSCAFT00000003710	F	5′-TCACTGGTGAGAACCCCCT-3′	62.5	141
		R	5′-CCTGATTCACACGGCGTAG-3′		
