# Supplementary material for: Aberrant hepatic lipid storage and metabolism in canine portosystemic shunts
Source: PLoS One. 2017 Oct 19;12(10):e0186491. doi: 10.1371/journal.pone.0186491 (PMC5648188; doi:10.1371/journal.pone.0186491)
Supplement: S2 Table — Top 24 list of most up and down regulated genes by gene-expression profiling of hepatic tissue of dogs with a congenital portosystemic shunt compared to healthy liver samples. Fold change of the microarray (MA) and quantitative reversed transcriptase PCR (RT-qPCR) are displayed. (PDF) [file pone.0186491.s007.pdf]

Gene	Ensemble Gene ID	Description	EH MA	IH MA	EH qPCR	IH qPCR
PLIN2	ENSCAFG00000001601	perilipin 2	2.47	2.03	1.82	5.24
ALDH1B1	ENSCAFG00000002400	aldehyde dehydrogenase 1 family member B1	-1.80	-1.82	NA	NA
MGAM	ENSCAFG00000003841	maltase-glucoamylase	-1.76	-1.78	NA	NA
FCGBP	ENSCAFG00000005406	Fc fragment of IgG binding protein	-1.48	-1.60	NA	NA
FABP1	ENSCAFG00000007413	fatty acid binding protein 1, liver	4.36	3.67	20.36	66.46
ELOVL2	ENSCAFG00000009756	ELOVL fatty acid elongase 2	2.17	1.84	N.A.	N.A.
ELOVL5	ENSCAFG00000002276	ELOVL fatty acid elongase 5	N.A.	N.A.	1.03	1.19
ELOVL6	ENSCAFG00000011549	ELOVL fatty acid elongase 6	N.A.	N.A.	1.29	-1.25
HSD3B2	ENSCAFG00000010039	3-beta-hydroxysteroid dehydrogenase/Delta 5-->4-isomerase	-2.21	-1.67	-4.18	-8.60
CRP	ENSCAFG00000011787	C-reactive protein, pentraxin-related	2.92	2.36	4.26	5.95
IGFBP1	ENSCAFG00000012272	insulin like growth factor binding protein 1	3.39	3.08	8.50	17.52
DMBT1	ENSCAFG00000012561	deleted in malignant brain tumors 1	-2.48	-2.63	NA	NA
SEC14L3	ENSCAFG00000012757	SEC14-like lipid binding 3	-1.53	-2.51	-2.35	-2.28
NNMT	ENSCAFG00000013528	Nicotinamide N-Methyltransferase	-3.74	-2.06	NA	NA
CBR1	ENSCAFG00000014444	Uncharacterized protein	-1.71	-1.89	NA	NA
ITIH3	ENSCAFG00000015068	inter-alpha-trypsin inhibitor heavy chain 3	2.35	1.87	1.73	1.77
SAA1	ENSCAFG00000015205	Serum amyloid A protein	2.04	1.01	4.43	12.99
RGS7	ENSCAFG00000015679	regulator of G-protein signaling 7	-1.74	-1.71	NA	NA
CYP1A2	ENSCAFG00000017941	cytochrome P450 family 1 subfamily A member 2	-2.20	-1.72	NA	NA
CCL4	ENSCAFG00000018164	Chemokine (C-C motif) ligand 4	-1.32	-1.61	NA	NA
GSTM3	ENSCAFG00000019809	glutathione S-transferase mu 3 (brain)	-2.21	-1.72	NA	NA
GSTM4	ENSCAFG00000019812	glutathione S-transferase mu 4	-1.62	-1.65	NA	NA
COX2	ENSCAFG00000022726	Cytochrome c oxidase subunit 2	-1.51	-1.65	NA	NA
CA3	ENSCAFG00000025237	carbonic anhydrase III	-3.52	-2.94	NA	NA
ITIH4	ENSCAFG00000025533	inter-alpha-trypsin inhibitor heavy chain family member 4	3.11	2.44	3.38	5.34
IL33	ENSCAFG00000030105	interleukin 33	-2.35	-1.98	NA	NA
