# Supplementary material for: Aberrant hepatic lipid storage and metabolism in canine portosystemic shunts
Source: PLoS One. 2017 Oct 19;12(10):e0186491. doi: 10.1371/journal.pone.0186491 (PMC5648188; doi:10.1371/journal.pone.0186491)
Supplement: S3 Table — (PDF) [file pone.0186491.s008.pdf]

	mz/Rt	polarity	CTR_1	CTR_2	CTR_3	CTR_4	CTR_5	EH_1	EH_2	EH_3	EH_4	EH_5	EH_6	IH_1	IH_2	IH_3	IH_4	IH_5	IH_6	IH_7
BMP 30:4	685.6/83.9	neg.	0.009	0.006	0.003	0.023	0.048	0.003	0.010	0.012	0.002	0.113	0.026	0.064	0.019	0.012	0.009	0.052	0.028	0.050
BMP 30:3	687.6/83.8	neg.	0.005	0.004	0.004	0.005	0.027	0.002	0.006	0.006	0.003	0.039	0.014	0.035	0.005	0.006	0.006	0.045	0.009	0.019
BMP A32:4	699.6/83	neg.	0.007	0.009	0.007	0.008	0.008	0.008	0.008	0.015	0.106	0.024	0.116	0.016	0.010	0.006	0.033	0.026	0.008	0.006
BMP A32:3	701.6/83	neg.	0.006	0.004	0.005	0.005	0.003	0.002	0.003	0.007	0.051	0.013	0.070	0.010	0.005	0.003	0.017	0.024	0.003	0.002
BMP 32:6	709.5/82.6	neg.	0.004	0.005	0.004	0.006	0.017	0.004	0.017	0.006	0.008	0.030	0.005	0.029	0.006	0.003	0.005	0.016	0.010	0.016
BMP 32:5	711.6/83.2	neg.	0.006	0.008	0.002	0.006	0.016	0.002	0.009	0.005	0.003	0.024	0.008	0.015	0.007	0.002	0.005	0.024	0.007	0.011
BMP 32:4	713.6/83.3	neg.	0.015	0.010	0.005	0.018	0.051	0.004	0.013	0.016	0.010	0.088	0.032	0.037	0.012	0.010	0.009	0.055	0.022	0.038
BMP 32:3	715.6/83.3	neg.	0.008	0.004	0.004	0.005	0.017	0.003	0.007	0.004	0.006	0.013	0.016	0.013	0.006	0.005	0.009	0.029	0.009	0.009
BMP A34:5	725.7/82.4	neg.	0.005	0.005	0.005	0.006	0.018	0.004	0.004	0.010	0.034	0.011	0.052	0.009	0.003	0.003	0.018	0.016	0.002	0.003
BMP A34:4	727.6/82.3	neg.	0.006	0.005	0.008	0.006	0.007	0.001	0.038	0.015	0.122	0.024	0.155	0.018	0.003	0.012	0.035	0.022	0.004	0.003
BMP 34:7	735.6/82.5	neg.	0.005	0.003	0.004	0.006	0.008	0.005	0.005	0.004	0.003	0.015	0.005	0.017	0.005	0.005	0.005	0.011	0.005	0.009
BMP 34:6	737.6/82.3	neg.	0.011	0.008	0.008	0.027	0.030	0.006	0.007	0.010	0.015	0.038	0.012	0.033	0.012	0.010	0.007	0.032	0.011	0.019
BMP 34:5	739.6/82.5	neg.	0.006	0.009	0.008	0.008	0.015	0.005	0.018	0.007	0.008	0.022	0.006	0.012	0.007	0.005	0.008	0.025	0.005	0.027
BMP 34:3	743.6/81.8	neg.	0.044	0.094	0.054	0.085	0.053	0.018	0.067	0.031	0.022	0.031	0.032	0.019	0.026	0.028	0.039	0.102	0.022	0.022
BMP A36:6	751.7/81.5	neg.	0.007	0.006	0.006	0.008	0.008	0.002	0.005	0.006	0.123	0.014	0.093	0.012	0.006	0.004	0.020	0.029	0.015	0.006
BMP 36:5	767.7/81.5	neg.	0.084	0.140	0.099	0.180	0.153	0.069	0.160	0.075	0.051	0.086	0.141	0.052	0.067	0.046	0.105	0.160	0.046	0.044
BMP 36:4	769.7/81.5	neg.	0.307	0.488	0.322	0.789	0.275	0.113	0.452	0.128	0.170	0.143	0.246	0.125	0.137	0.118	0.199	0.386	0.113	0.121
BMP 36:3	771.7/81.4	neg.	0.790	0.864	0.827	1.404	0.641	0.306	1.120	0.346	0.420	0.375	0.659	0.317	0.327	0.465	0.627	0.986	0.446	0.362
BMP 38:9	787.7/81.1	neg.	0.017	0.023	0.017	0.012	0.000	0.008	0.051	0.023	0.028	0.012	0.022	0.050	0.017	0.015	0.055	0.034	0.004	0.020
PG 30:6	681.6/89.7	neg.	0.008	0.007	0.010	0.004	0.018	0.001	0.002	0.001	0.002	0.001	0.021	0.001	0.001	0.001	0.002	0.005	0.001	0.001
PG 30:0	693.6/89.1	neg.	0.015	0.015	0.023	0.044	0.012	0.003	0.085	0.006	0.005	0.009	0.013	0.005	0.007	0.004	0.011	0.022	0.004	0.003
PG 32:6	709.6/89.3	neg.	0.013	0.022	0.018	0.010	0.023	0.001	0.003	0.001	0.000	0.000	0.015	0.003	0.001	0.001	0.004	0.003	0.001	0.000
PG 32:1	719.6/88.5	neg.	0.035	0.042	0.025	0.012	0.017	0.009	0.013	0.013	0.009	0.006	0.016	0.007	0.009	0.009	0.037	0.048	0.012	0.002
PG 32:0	721.6/88.8	neg.	0.030	0.025	0.020	0.015	0.025	0.012	0.015	0.022	0.024	0.017	0.012	0.031	0.022	0.022	0.022	0.057	0.034	0.014
PG 34:2	745.7/87.9	neg.	0.220	0.279	0.242	0.233	0.175	0.139	0.272	0.198	0.242	0.205	0.169	0.161	0.234	0.145	0.369	0.243	0.217	0.164
PG 34:1	747.6/88.2	neg.	1.054	1.188	1.105	1.056	1.075	1.195	1.072	1.132	1.165	1.430	1.260	1.331	1.705	1.264	0.817	1.005	1.453	1.389
PG 34:0	749.6/88.2	neg.	0.064	0.041	0.039	0.051	0.045	0.040	0.071	0.060	0.055	0.078	0.051	0.052	0.059	0.059	0.043	0.049	0.055	0.051
PG 36:8	761.6/87.9	neg.	0.066	0.042	0.051	0.052	0.063	0.052	0.050	0.059	0.051	0.086	0.073	0.041	0.056	0.089	0.066	0.098	0.083	0.041
PG 36:2	773.7/87.1	neg.	0.894	0.742	0.821	0.799	0.818	0.637	1.414	0.830	0.726	0.716	0.709	0.685	0.740	1.013	1.104	0.814	0.720	0.735
PG 36:1	775.7/87.5	neg.	0.431	0.329	0.382	0.413	0.457	0.655	0.435	0.591	0.557	0.624	0.643	0.858	0.725	0.506	0.367	0.489	0.616	0.617
PG 36:0	777.6/87.5	neg.	0.017	0.022	0.026	0.026	0.033	0.003	0.041	0.032	0.327	0.016	0.207	0.026	0.031	0.023	0.030	0.020	0.028	0.024
PG 38:4	797.7/86.1	neg.	0.289	0.239	0.273	0.394	0.294	0.263	0.274	0.321	0.300	0.342	0.240	0.355	0.262	0.326	0.295	0.332	0.303	0.277
PG 38:2	801.6/87	neg.	0.122	0.105	0.079	0.136	0.105	0.157	0.116	0.129	0.142	0.116	0.131	0.168	0.120	0.114	0.102	0.099	0.116	0.115
PG 38:0	805.6/88.7	neg.	0.023	0.036	0.048	0.038	0.031	0.100	0.028	0.112	0.319	0.041	0.161	0.067	0.063	0.039	0.038	0.037	0.038	0.067
PI 30:0	781.6/99.5	neg.	0.067	0.057	0.051	0.032	0.070	0.024	0.039	0.021	0.018	0.022	0.048	0.018	0.039	0.023	0.054	0.084	0.041	0.034
PI 32:1	807.7/98.2	neg.	0.045	0.062	0.045	0.030	0.080	0.026	0.060	0.024	0.019	0.022	0.074	0.028	0.057	0.034	0.085	0.140	0.050	0.035
PI 32:0	809.7/98.7	neg.	0.117	0.166	0.093	0.092	0.160	0.115	0.199	0.090	0.106	0.090	0.231	0.119	0.157	0.097	0.193	0.307	0.193	0.098
PI 34:7	823.7/98.8	neg.	0.022	0.018	0.016	0.015	0.022	0.029	0.028	0.022	0.016	0.031	0.033	0.011	0.021	0.041	0.039	0.138	0.041	0.013

PI 34:2	833.7/97.8	neg.	0.350	0.449	0.388	0.420	0.486	0.193	0.849	0.219	0.312	0.230	0.806	0.261	0.337	0.216	1.042	0.480	0.349	0.300
PI 34:1	835.7/98.1	neg.	0.483	0.474	0.468	0.344	0.637	0.366	0.725	0.291	0.355	0.387	0.950	0.432	0.484	0.419	0.917	1.067	0.612	0.524
PI 34:0	837.7/98.5	neg.	0.169	0.208	0.127	0.167	0.205	0.148	0.209	0.126	0.120	0.108	0.217	0.124	0.189	0.168	0.191	0.311	0.239	0.163
PI A36:6	839.7/98.5	neg.	0.006	0.007	0.008	0.007	0.009	0.004	0.010	0.003	0.004	0.011	0.008	0.004	0.007	0.004	0.004	0.013	0.010	0.006
PI 36:9	847.7/97.9	neg.	0.047	0.035	0.043	0.028	0.041	0.014	0.057	0.026	0.019	0.019	0.050	0.012	0.017	0.044	0.104	0.075	0.030	0.019
PI 36:8	849.7/98.1	neg.	0.067	0.056	0.055	0.040	0.069	0.056	0.070	0.050	0.037	0.059	0.071	0.040	0.049	0.090	0.121	0.179	0.069	0.058
PI 36:7	851.7/98.5	neg.	0.012	0.009	0.004	0.006	0.016	0.005	0.020	0.007	0.006	0.018	0.021	0.004	0.015	0.029	0.017	0.065	0.025	0.006
PI 36:4	857.8/96.2	neg.	0.290	0.352	0.329	0.396	0.374	0.351	0.396	0.190	0.206	0.230	0.294	0.313	0.288	0.132	0.318	0.348	0.233	0.252
PI 36:3	859.7/96.9	neg.	0.212	0.170	0.259	0.189	0.206	0.066	0.235	0.112	0.114	0.101	0.272	0.068	0.143	0.134	0.604	0.423	0.168	0.136
PI 36:2	861.7/97.3	neg.	0.983	0.914	1.153	0.977	1.250	0.288	1.432	0.459	0.482	0.467	1.620	0.357	0.698	0.800	1.693	1.310	0.863	0.665
PI 36:1	863.7/97.8	neg.	0.612	0.578	0.554	0.471	0.750	0.541	0.727	0.647	0.573	0.764	0.914	0.733	0.734	1.081	0.878	1.138	1.101	1.018
PI 36:0	865.7/98.2	neg.	0.123	0.100	0.111	0.263	0.262	0.045	0.092	0.074	0.079	0.089	0.089	0.075	0.058	0.118	0.114	0.010	0.112	0.085
PI A38:6	867.7/98.8	neg.	0.009	0.013	0.006	0.017	0.012	0.005	0.006	0.002	0.001	0.001	0.003	0.005	0.004	0.003	0.005	0.029	0.006	0.002
PI A38:4	871.8/95.9	neg.	0.074	0.045	0.066	0.050	0.065	0.081	0.050	0.038	0.038	0.039	0.038	0.039	0.030	0.062	0.057	0.096	0.046	0.030
PI 38:10	873.7/96.5	neg.	0.015	0.005	0.011	0.014	0.010	0.013	0.026	0.017	0.010	0.010	0.002	0.010	0.009	0.026	0.008	0.042	0.030	0.007
PI 38:9	875.7/97.8	neg.	0.023	0.020	0.027	0.014	0.018	0.013	0.013	0.012	0.006	0.009	0.020	0.008	0.007	0.016	0.040	0.038	0.014	0.008
PI 38:6	881.8/95.6	neg.	0.044	0.043	0.055	0.030	0.043	0.058	0.110	0.073	0.059	0.073	0.105	0.079	0.072	0.042	0.096	0.074	0.104	0.070
PI 38:5	883.8/95.3	neg.	0.497	0.569	0.638	0.281	0.476	0.272	0.273	0.217	0.243	0.180	0.331	0.232	0.264	0.251	0.411	1.095	0.267	0.209
PI 38:4	885.8/95.6	neg.	4.417	4.869	5.137	5.622	5.907	6.468	4.659	4.757	4.773	5.140	3.380	5.644	4.675	4.381	2.743	3.350	3.574	4.824
PI 38:3	887.7/96.1	neg.	0.706	0.698	0.662	0.851	0.616	1.214	1.048	1.467	1.229	1.414	0.491	1.147	0.916	1.353	0.396	0.764	1.070	0.851
PI 38:2	889.7/97.1	neg.	0.143	0.167	0.113	0.230	0.137	0.185	0.147	0.255	0.205	0.248	0.134	0.198	0.169	0.298	0.088	0.187	0.217	0.170
PI 38:1	891.7/97.5	neg.	0.026	0.019	0.022	0.017	0.020	0.030	0.017	0.038	0.030	0.030	0.017	0.025	0.029	0.063	0.028	0.039	0.037	0.017
PI 38:0	893.6/97	neg.	0.008	0.009	0.009	0.006	0.004	0.035	0.020	0.016	0.014	0.002	0.002	0.001	0.028	0.013	0.022	0.004	0.001	0.004
PI A40:4	899.6/95.1	neg.	0.069	0.081	0.085	0.039	0.067	0.051	0.039	0.052	0.106	0.026	0.027	0.020	0.022	0.039	0.038	0.073	0.025	0.032
PI 40:10	901.6/97.2	neg.	0.050	0.062	0.039	0.027	0.047	0.060	0.042	0.086	0.056	0.020	0.019	0.064	0.027	0.028	0.038	0.035	0.019	0.021
PI 40:9	903.6/97.7	neg.	0.011	0.008	0.008	0.005	0.009	0.011	0.009	0.039	0.019	0.004	0.002	0.011	0.007	0.004	0.011	0.008	0.006	0.006
PI 40:7	907.6/94.5	neg.	0.017	0.014	0.003	0.000	0.012	0.001	0.007	0.012	0.000	0.014	0.013	0.006	0.001	0.006	0.037	0.038	0.048	0.001
PI 40:6	909.7/94.9	neg.	0.271	0.280	0.292	0.124	0.241	0.310	0.344	0.515	0.406	0.448	0.279	0.482	0.499	0.593	0.209	0.213	0.634	0.506
PI 40:5	911.7/95.2	neg.	0.474	0.510	0.622	0.243	0.467	0.354	0.321	0.311	0.292	0.245	0.168	0.322	0.352	0.349	0.170	0.310	0.368	0.300
PI 40:4	913.7/95.6	neg.	0.180	0.268	0.263	0.128	0.189	0.158	0.176	0.122	0.132	0.120	0.081	0.134	0.165	0.125	0.087	0.169	0.131	0.147
PI 40:3	915.7/95.8	neg.	0.034	0.013	0.042	0.038	0.009	0.014	0.011	0.038	0.012	0.010	0.009	0.008	0.013	0.027	0.020	0.018	0.027	0.016
PI 40:2	917.6/94.7	neg.	0.025	0.036	0.018	0.092	0.023	0.006	0.007	0.033	0.008	0.036	0.015	0.012	0.029	0.003	0.028	0.009	0.035	0.008
PI 40:1	919.6/98.8	neg.	0.012	0.009	0.011	0.022	0.026	0.006	0.012	0.036	0.013	0.006	0.004	0.016	0.006	0.006	0.010	0.003	0.009	0.008
PI 40:0	921.4/99	neg.	0.004	0.005	0.003	0.004	0.006	0.006	0.007	0.005	0.003	0.001	0.003	0.004	0.008	0.004	0.003	0.002	0.003	0.009
PI A42:6	923.6/94.6	neg.	0.010	0.015	0.011	0.003	0.008	0.007	0.008	0.009	0.011	0.016	0.005	0.019	0.006	0.019	0.008	0.009	0.010	0.010
PI 42:9	931.6/98.8	neg.	0.007	0.009	0.006	0.024	0.006	0.006	0.005	0.021	0.027	0.005	0.003	0.010	0.008	0.003	0.014	0.003	0.011	0.005
PE 32:2	686.6/104.5	neg.	0.011	0.027	0.017	0.008	0.016	0.001	0.011	0.011	0.004	0.003	0.004	0.003	0.008	0.006	0.017	0.019	0.007	0.006
PE 32:1	688.6/104.2	neg.	0.036	0.051	0.040	0.033	0.044	0.024	0.032	0.040	0.021	0.027	0.030	0.018	0.051	0.028	0.040	0.107	0.035	0.035
PE 32:0	690.6/104.3	neg.	0.008	0.004	0.001	0.000	0.003	0.000	0.002	0.005	0.001	0.001	0.002	0.005	0.004	0.005	0.003	0.006	0.000	0.001

PE A34:3	698.7/102.7	neg.	0.023	0.023	0.024	0.020	0.012	0.009	0.025	0.018	0.017	0.014	0.017	0.007	0.011	0.015	0.016	0.026	0.012	0.013
PE A34:2	700.7/103	neg.	0.020	0.030	0.032	0.022	0.028	0.010	0.041	0.017	0.015	0.014	0.017	0.018	0.019	0.022	0.021	0.033	0.021	0.024
PE 34:8	702.6/103.9	neg.	0.021	0.013	0.015	0.023	0.009	0.006	0.010	0.010	0.004	0.004	0.014	0.004	0.010	0.020	0.018	0.042	0.009	0.009
PE 34:4	710.6/103.2	neg.	0.005	0.012	0.009	0.005	0.006	0.000	0.003	0.004	0.002	0.002	0.003	0.001	0.003	0.002	0.003	0.010	0.004	0.003
PE 34:3	712.6/103.8	neg.	0.039	0.089	0.045	0.061	0.052	0.017	0.045	0.035	0.024	0.032	0.060	0.014	0.030	0.027	0.052	0.092	0.038	0.028
PE 34:2	714.6/103.6	neg.	0.470	0.748	0.509	0.973	0.664	0.611	0.983	0.783	0.698	0.915	0.996	0.500	0.833	0.586	0.993	0.440	0.744	0.628
PE 34:1	716.6/103.7	neg.	0.338	0.306	0.306	0.286	0.417	0.284	0.329	0.231	0.266	0.186	0.412	0.191	0.262	0.244	0.466	0.516	0.221	0.284
PE 34:0	718.7/103.9	neg.	0.006	0.015	0.008	0.013	0.025	0.016	0.016	0.012	0.013	0.014	0.010	0.009	0.009	0.008	0.013	0.024	0.009	0.012
PE A36:6	720.7/101.6	neg.	0.004	0.006	0.003	0.006	0.006	0.003	0.011	0.013	0.008	0.010	0.012	0.013	0.009	0.012	0.009	0.011	0.012	0.005
PE A36:5	722.7/101.3	neg.	0.127	0.136	0.177	0.144	0.099	0.098	0.108	0.112	0.097	0.111	0.098	0.137	0.160	0.134	0.099	0.132	0.136	0.140
PE A36:4	724.8/101.9	neg.	0.027	0.033	0.037	0.041	0.034	0.008	0.041	0.028	0.031	0.024	0.025	0.017	0.027	0.026	0.021	0.024	0.025	0.022
PE A36:3	726.8/102.3	neg.	0.040	0.039	0.049	0.051	0.029	0.031	0.063	0.057	0.051	0.058	0.057	0.031	0.055	0.062	0.068	0.065	0.028	0.030
PE 36:9	728.7/103.5	neg.	0.073	0.072	0.090	0.080	0.072	0.065	0.115	0.085	0.057	0.086	0.117	0.041	0.066	0.091	0.132	0.103	0.070	0.069
PE 36:8	730.7/104.2	neg.	0.079	0.035	0.050	0.035	0.073	0.033	0.023	0.036	0.027	0.024	0.066	0.017	0.024	0.032	0.080	0.085	0.026	0.025
PE 36:6	734.7/102.5	neg.	0.006	0.016	0.011	0.003	0.012	0.003	0.008	0.005	0.002	0.005	0.006	0.004	0.004	0.007	0.006	0.009	0.011	0.008
PE 36:5	736.7/102.2	neg.	0.063	0.080	0.064	0.052	0.073	0.031	0.053	0.083	0.045	0.061	0.093	0.037	0.043	0.075	0.056	0.104	0.081	0.045
PE 36:4	738.7/102.3	neg.	0.640	0.876	0.714	0.972	0.801	0.892	0.662	1.111	1.110	1.245	1.044	0.893	0.959	0.465	0.940	0.541	1.094	0.881
PE 36:3	740.7/102.9	neg.	0.426	0.305	0.518	0.446	0.367	0.127	0.225	0.180	0.135	0.209	0.427	0.115	0.140	0.233	0.494	0.470	0.250	0.206
PE 36:2	742.7/103	neg.	1.294	0.914	0.950	1.432	1.060	0.628	0.983	0.966	0.676	0.945	1.353	0.435	0.993	0.869	1.315	0.844	0.658	0.742
PE 36:1	744.7/103	neg.	0.427	0.178	0.283	0.259	0.381	0.183	0.174	0.184	0.158	0.159	0.351	0.158	0.210	0.208	0.275	0.299	0.264	0.151
PE 36:0	746.7/100.8	neg.	0.092	0.060	0.070	0.056	0.067	0.048	0.075	0.073	0.067	0.067	0.099	0.104	0.074	0.065	0.119	0.054	0.076	0.105
PE A38:6	748.7/100.6	neg.	0.217	0.256	0.243	0.211	0.178	0.195	0.200	0.217	0.082	0.186	0.211	0.238	0.201	0.263	0.189	0.207	0.210	0.217
PE A38:5	750.7/100.5	neg.	0.376	0.339	0.464	0.457	0.310	0.484	0.387	0.449	0.471	0.428	0.483	0.433	0.479	0.417	0.449	0.650	0.408	0.453
PE A38:4	752.7/101.4	neg.	0.116	0.041	0.113	0.102	0.075	0.094	0.063	0.100	0.078	0.120	0.102	0.067	0.084	0.035	0.110	0.174	0.096	0.078
PE 38:7	760.7/101.9	neg.	0.022	0.030	0.033	0.010	0.025	0.016	0.023	0.022	0.014	0.025	0.026	0.018	0.017	0.037	0.019	0.028	0.026	0.028
PE 38:6	762.7/102	neg.	0.378	0.642	0.395	0.268	0.427	1.339	1.113	1.656	1.313	1.531	1.189	1.885	1.363	1.366	0.604	0.135	1.309	1.460
PE 38:5	764.8/102	neg.	0.798	0.907	0.874	0.490	0.895	0.445	0.440	0.368	0.466	0.409	0.626	0.449	0.419	0.491	0.496	0.760	0.592	0.372
PE 38:4	766.7/101.7	neg.	3.205	3.092	2.942	3.622	2.862	2.736	1.897	3.189	3.008	2.990	3.041	2.625	2.973	2.733	3.254	2.700	2.741	2.424
PE 38:3	768.6/101.9	neg.	0.176	0.142	0.149	0.091	0.097	0.170	0.180	0.128	0.117	0.224	0.180	0.109	0.169	0.170	0.161	0.166	0.193	0.099
PE 38:2	770.6/102.5	neg.	0.061	0.037	0.046	0.062	0.036	0.005	0.029	0.042	0.055	0.016	0.049	0.026	0.048	0.044	0.052	0.036	0.046	0.048
PE 38:1	772.6/100.1	neg.	0.036	0.031	0.031	0.028	0.028	0.033	0.059	0.050	0.042	0.044	0.050	0.055	0.067	0.065	0.036	0.017	0.049	0.083
PE 38:0	774.6/100.2	neg.	0.081	0.088	0.098	0.066	0.073	0.132	0.165	0.172	0.153	0.141	0.142	0.185	0.172	0.176	0.115	0.062	0.153	0.237
PE A40:6	776.6/100.6	neg.	0.138	0.128	0.153	0.116	0.120	0.166	0.126	0.143	0.145	0.139	0.137	0.150	0.190	0.183	0.108	0.166	0.163	0.183
PE A40:5	778.6/100.7	neg.	0.147	0.132	0.149	0.124	0.107	0.085	0.111	0.098	0.084	0.107	0.108	0.112	0.130	0.107	0.099	0.170	0.110	0.105
PE A40:4	780.6/102.3	neg.	0.108	0.085	0.093	0.064	0.080	0.043	0.009	0.061	0.038	0.041	0.043	0.050	0.042	0.050	0.090	0.062	0.012	0.058
PE 40:7	788.6/101.1	neg.	0.112	0.103	0.130	0.032	0.093	0.073	0.086	0.107	0.090	0.117	0.138	0.090	0.087	0.121	0.119	0.076	0.140	0.153
PE 40:6	790.6/101.2	neg.	0.475	0.526	0.500	0.240	0.475	1.012	0.813	1.359	1.067	1.238	0.680	1.382	1.000	0.967	0.447	0.196	0.991	1.255
PE 40:5	792.6/101.5	neg.	0.452	0.436	0.515	0.249	0.395	0.279	0.228	0.182	0.237	0.216	0.194	0.303	0.433	0.278	0.186	0.221	0.332	0.309
PE 40:4	794.6/101.8	neg.	0.174	0.225	0.269	0.091	0.147	0.086	0.084	0.088	0.091	0.076	0.072	0.078	0.111	0.079	0.102	0.115	0.086	0.064

PE 40:3	796.6/101.9	neg.	0.030	0.026	0.025	0.031	0.021	0.060	0.015	0.028	0.015	0.013	0.017	0.011	0.020	0.010	0.029	0.026	0.020	0.021
PS 34:2	758.5/126.2	neg.	0.215	0.294	0.253	0.293	0.238	0.106	0.337	0.213	0.298	0.175	0.396	0.195	0.272	0.247	0.331	0.038	0.266	0.206
PS 34:1	760.6/126	neg.	0.286	0.337	0.355	0.322	0.233	0.160	0.294	0.212	0.285	0.174	0.259	0.248	0.257	0.259	0.448	0.458	0.265	0.225
PS 34:0	762.5/128.2	neg.	0.048	0.062	0.054	0.046	0.042	0.021	0.061	0.044	0.068	0.047	0.061	0.335	0.062	0.071	0.070	0.093	0.069	0.055
PS 36:9	772.6/125	neg.	0.053	0.067	0.052	0.055	0.043	0.015	0.062	0.043	0.041	0.065	0.073	0.042	0.035	0.063	0.123	0.116	0.070	0.033
PS 36:8	774.6/125.9	neg.	0.034	0.036	0.052	0.041	0.028	0.026	0.046	0.076	0.025	0.053	0.085	0.042	0.047	0.189	0.084	0.109	0.053	0.031
PS 36:3	784.6/122.7	neg.	0.125	0.161	0.214	0.164	0.142	0.085	0.282	0.196	0.211	0.173	0.434	0.151	0.171	0.256	0.303	0.412	0.259	0.153
PS 36:2	786.6/122.4	neg.	2.689	2.511	2.472	3.303	2.204	1.843	4.162	2.740	2.837	2.822	5.818	2.553	3.154	3.199	2.925	3.003	3.068	3.442
PS 36:1	788.6/123.6	neg.	1.151	1.160	1.378	0.982	1.465	0.663	1.266	1.398	0.907	1.168	1.090	1.547	1.455	1.863	1.710	2.192	1.417	1.140
PS 36:0	790.5/125.2	neg.	0.002	0.109	0.029	0.024	0.048	0.063	0.135	0.097	0.080	0.107	0.047	0.147	0.088	0.140	0.045	0.054	0.093	0.051
PS 38:6	806.5/118.6	neg.	0.361	0.483	0.523	0.290	0.479	0.720	0.942	0.767	0.843	1.113	1.141	0.654	0.427	0.533	0.777	0.187	0.531	0.989
PS 38:5	808.5/118.8	neg.	0.513	0.871	0.505	0.678	0.876	0.333	0.464	0.444	0.404	0.199	0.608	0.314	0.478	0.581	0.490	0.644	0.319	0.199
PS 38:4	810.5/117.8	neg.	5.998	6.409	5.554	8.127	5.920	5.521	2.717	4.510	6.316	4.598	4.921	4.662	3.321	5.407	4.502	5.124	4.287	4.721
PS 38:3	812.5/119.3	neg.	0.718	1.480	1.517	2.029	1.193	1.005	1.629	1.638	2.001	1.304	0.950	1.568	1.397	1.613	3.014	1.370	2.539	1.024
PS 38:2	814.5/120.6	neg.	0.634	0.513	0.362	0.421	0.415	0.227	0.694	0.267	0.419	0.307	0.337	0.223	0.355	0.654	0.457	0.388	0.534	0.507
PS 38:1	816.6/125.3	neg.	0.074	0.221	0.276	0.109	0.197	0.082	0.205	0.175	0.273	0.210	0.182	0.263	0.190	0.253	0.426	0.444	0.186	0.148
PS A40:4	824.5/129.3	neg.	0.974	0.683	1.075	1.075	0.905	0.195	0.759	0.261	0.215	0.728	0.284	0.316	0.405	0.507	0.527	0.607	0.438	0.556
PS 40:10	826.5/129.2	neg.	0.031	0.045	0.051	0.069	0.059	0.013	0.029	0.206	0.091	0.056	0.045	0.113	0.029	0.045	0.088	0.086	0.041	0.149
PS 40:7	832.5/118.2	neg.	0.074	0.124	0.125	0.114	0.121	0.196	0.135	0.174	0.242	0.032	0.137	0.088	0.136	0.168	0.167	0.264	0.215	0.143
PS 40:6	834.5/116.5	neg.	2.089	3.446	2.879	1.806	3.639	8.364	6.043	9.183	7.609	9.619	4.381	9.845	4.371	6.344	4.203	2.273	7.194	8.378
PS 40:5	836.5/117.6	neg.	5.385	4.307	4.966	2.943	5.088	2.539	2.784	2.135	2.599	2.286	1.804	3.021	3.358	3.072	2.836	4.465	2.634	2.549
PS 40:4	838.5/117.6	neg.	2.131	2.221	2.799	1.230	1.961	1.050	1.245	0.769	1.084	0.999	1.143	1.083	1.375	1.228	1.993	2.359	1.364	1.311
PS 40:3	840.5/119.7	neg.	0.357	0.554	0.649	0.186	0.410	0.336	0.322	0.321	0.283	0.347	0.369	0.281	0.255	0.332	0.479	0.272	0.427	0.324
PS 40:2	842.5/122.8	neg.	0.174	0.237	0.168	0.123	0.130	0.055	0.175	0.119	0.106	0.135	0.102	0.121	0.116	0.199	0.124	0.220	0.111	0.117
PS 40:1	844.5/124.4	neg.	0.144	0.190	0.153	0.114	0.137	0.057	0.163	0.240	0.173	0.118	0.136	0.154	0.104	0.130	0.113	0.209	0.090	0.091
PS 40:0	846.4/126.2	neg.	0.042	0.042	0.044	0.034	0.037	0.041	0.048	0.047	0.034	0.056	0.040	0.037	0.044	0.043	0.047	0.061	0.040	0.052
PS A42:6	848.5/128.3	neg.	0.105	0.103	0.123	0.051	0.131	0.102	0.112	0.067	0.048	0.087	0.075	0.055	0.061	0.082	0.065	0.116	0.117	0.230
lysoPE C16:1	450.4/133.3	neg.	0.009	0.014	0.011	0.007	0.009	0.003	0.005	0.012	0.006	0.007	0.006	0.004	0.006	0.013	0.008	0.011	0.015	0.005
lysoPE C16:0	452.4/132.2	neg.	0.477	0.668	0.543	0.615	0.470	1.062	0.890	1.575	1.541	1.494	0.526	1.233	1.557	1.329	0.733	0.299	1.596	1.037
lysoPE C18:1	478.4/129.7	neg.	0.229	0.140	0.205	0.158	0.148	0.064	0.083	0.155	0.132	0.137	0.127	0.062	0.106	0.109	0.185	0.192	0.168	0.090
lysoPE C18:0	480.4/128.7	neg.	1.109	0.877	0.799	1.584	0.825	1.154	0.964	1.720	2.157	1.520	0.787	1.153	1.623	1.192	1.244	0.606	1.473	1.015
lysoPE C22:5	526.2/132.3	neg.	0.017	0.015	0.014	0.014	0.015	0.044	0.027	0.033	0.024	0.024	0.017	0.021	0.029	0.020	0.015	0.008	0.026	0.031
lysoPE C22:4	528.2/132.3	neg.	0.004	0.004	0.005	0.005	0.004	0.018	0.013	0.010	0.008	0.006	0.006	0.006	0.012	0.009	0.005	0.002	0.009	0.011
lysoPE C22:0	536.3/130.2	neg.	0.014	0.007	0.009	0.008	0.010	0.008	0.007	0.008	0.008	0.005	0.008	0.005	0.007	0.008	0.011	0.007	0.009	0.008
lysoPE C24:5	554.2/128.9	neg.	0.015	0.011	0.011	0.011	0.013	0.032	0.016	0.018	0.017	0.013	0.014	0.012	0.028	0.014	0.017	0.005	0.018	0.029
lysoPE C24:4	556.2/129	neg.	0.006	0.004	0.001	0.004	0.006	0.013	0.007	0.011	0.006	0.005	0.003	0.007	0.006	0.008	0.006	0.002	0.007	0.008
lysoPE C26:5	582.2/132.2	neg.	0.003	0.004	0.004	0.003	0.003	0.011	0.006	0.007	0.012	0.007	0.004	0.005	0.007	0.004	0.005	0.001	0.007	0.008
PC 28:0	678.7/157.6	pos.	0.017	0.007	0.013	0.005	0.002	0.005	0.004	0.011	0.000	0.003	0.012	0.015	0.002	0.006	0.000	0.021	0.003	0.002
PC A30:1	690.7/154	pos.	0.001	0.002	0.003	0.003	0.002	0.002	0.002	0.002	0.002	0.001	0.002	0.004	0.003	0.002	0.003	0.011	0.005	0.004

PC 30:7	692.7/156.5	pos.	0.012	0.012	0.016	0.010	0.008	0.014	0.011	0.013	0.009	0.008	0.010	0.027	0.012	0.028	0.011	0.044	0.036	0.011
PC 30:6	694.7/156.5	pos.	0.001	0.001	0.001	0.000	0.000	0.000	0.000	0.001	0.001	0.001	0.001	0.001	0.000	0.001	0.001	0.002	0.001	0.001
PC 30:2	702.7/154.9	pos.	0.003	0.003	0.006	0.004	0.003	0.003	0.003	0.003	0.004	0.003	0.006	0.009	0.006	0.003	0.008	0.010	0.006	0.002
PC 30:1	704.7/154.6	pos.	0.047	0.044	0.056	0.015	0.027	0.011	0.009	0.012	0.008	0.011	0.010	0.029	0.010	0.019	0.026	0.104	0.022	0.013
PC 30:0	706.7/155.1	pos.	0.166	0.102	0.150	0.059	0.101	0.084	0.049	0.091	0.058	0.057	0.096	0.152	0.070	0.092	0.076	0.303	0.119	0.060
PC A32:2	716.7/153.6	pos.	0.004	0.002	0.005	0.003	0.002	0.002	0.003	0.002	0.003	0.001	0.002	0.003	0.002	0.006	0.003	0.009	0.009	0.003
PC 32:8	718.7/153.2	pos.	0.047	0.036	0.065	0.046	0.036	0.043	0.041	0.024	0.036	0.029	0.039	0.052	0.045	0.088	0.035	0.092	0.098	0.072
PC 32:7	720.7/154.3	pos.	0.149	0.137	0.228	0.154	0.100	0.210	0.153	0.148	0.129	0.111	0.184	0.216	0.236	0.381	0.137	0.253	0.442	0.234
PC 32:6	722.7/154.6	pos.	0.002	0.006	0.008	0.006	0.005	0.011	0.008	0.005	0.002	0.004	0.006	0.006	0.004	0.006	0.005	0.010	0.014	0.014
PC 32:3	728.7/153.5	pos.	0.020	0.014	0.026	0.007	0.013	0.003	0.004	0.005	0.003	0.004	0.004	0.009	0.003	0.005	0.007	0.024	0.008	0.008
PC 32:2	730.7/152.6	pos.	0.248	0.285	0.293	0.098	0.166	0.046	0.068	0.080	0.059	0.071	0.071	0.129	0.051	0.105	0.153	0.177	0.109	0.095
PC 32:1	732.7/152.6	pos.	0.636	0.483	0.530	0.300	0.430	0.294	0.229	0.218	0.232	0.349	0.262	0.483	0.184	0.416	0.420	0.955	0.391	0.374
PC 32:0	734.7/153.2	pos.	0.617	0.653	0.753	0.717	0.516	0.889	0.667	0.872	0.761	0.594	1.153	0.889	1.100	0.742	0.663	0.712	0.921	0.900
PC A34:5	738.7/153.2	pos.	0.001	0.000	0.000	0.000	0.000	0.000	0.000	0.002	0.000	0.000	0.000	0.000	0.000	0.000	0.000	0.000	0.000	0.000
PC A34:3	742.7/146.8	pos.	0.005	0.005	0.008	0.013	0.005	0.005	0.006	0.005	0.004	0.004	0.003	0.004	0.004	0.004	0.015	0.018	0.004	0.004
PC A34:2	744.7/152	pos.	0.098	0.093	0.093	0.076	0.086	0.067	0.093	0.076	0.068	0.067	0.062	0.059	0.087	0.107	0.093	0.090	0.101	0.083
PC 34:8	746.7/152.2	pos.	0.193	0.122	0.179	0.146	0.155	0.161	0.187	0.168	0.144	0.139	0.163	0.168	0.201	0.313	0.175	0.365	0.264	0.210
PC 34:7	748.7/152.5	pos.	0.056	0.022	0.054	0.054	0.040	0.069	0.067	0.087	0.080	0.056	0.057	0.066	0.089	0.124	0.090	0.173	0.099	0.060
PC 34:6	750.7/152.8	pos.	0.002	0.001	0.002	0.001	0.000	0.004	0.002	0.003	0.004	0.000	0.001	0.000	0.005	0.001	0.003	0.006	0.004	0.001
PC 34:4	754.7/146.9	pos.	0.083	0.087	0.086	0.043	0.065	0.016	0.017	0.018	0.008	0.015	0.023	0.030	0.017	0.038	0.039	0.063	0.034	0.027
PC 34:3	756.7/149.5	pos.	0.370	0.656	0.536	0.291	0.371	0.183	0.263	0.164	0.215	0.300	0.173	0.232	0.196	0.263	0.361	0.472	0.260	0.285
PC 34:2	758.7/150.6	pos.	3.425	4.198	3.394	4.636	3.428	4.418	5.695	3.866	4.686	4.700	4.848	5.925	4.795	4.747	6.639	3.062	4.395	4.766
PC 34:1	760.7/150.9	pos.	3.328	2.519	2.751	2.564	3.101	3.339	2.494	2.172	2.518	2.820	3.172	3.222	2.804	3.008	3.840	3.776	2.836	2.810
PC 34:0	762.6/151.5	pos.	0.168	0.129	0.143	0.189	0.139	0.193	0.190	0.212	0.202	0.167	0.200	0.211	0.247	0.190	0.193	0.228	0.220	0.218
PC A36:6	764.7/151.8	pos.	0.008	0.006	0.005	0.007	0.006	0.008	0.000	0.012	0.015	0.004	0.012	0.013	0.006	0.010	0.019	0.011	0.012	0.011
PC A36:5	766.7/140.7	pos.	0.029	0.026	0.032	0.056	0.031	0.038	0.041	0.033	0.028	0.041	0.025	0.040	0.035	0.031	0.074	0.094	0.027	0.037
PC A36:4	768.7/145.6	pos.	0.106	0.096	0.105	0.166	0.091	0.135	0.099	0.104	0.080	0.092	0.113	0.156	0.157	0.168	0.132	0.177	0.176	0.188
PC A36:3	770.7/147.7	pos.	0.091	0.078	0.089	0.080	0.064	0.077	0.118	0.078	0.044	0.090	0.055	0.063	0.092	0.088	0.076	0.133	0.012	0.084
PC 36:9	772.7/149.1	pos.	0.216	0.177	0.195	0.168	0.173	0.112	0.159	0.160	0.157	0.192	0.096	0.107	0.136	0.231	0.252	0.292	0.172	0.125
PC 36:8	774.7/150.4	pos.	0.180	0.080	0.123	0.086	0.133	0.136	0.085	0.093	0.095	0.106	0.076	0.078	0.095	0.157	0.159	0.256	0.132	0.078
PC 36:7	776.6/151.3	pos.	0.014	0.061	0.039	0.008	0.016	0.029	0.024	0.014	0.018	0.021	0.085	0.008	0.014	0.019	0.018	0.052	0.016	0.013
PC 36:6	778.7/145.3	pos.	0.022	0.026	0.027	0.003	0.024	0.002	0.008	0.009	0.008	0.017	0.015	0.014	0.008	0.022	0.016	0.027	0.020	0.021
PC 36:5	780.7/144.9	pos.	0.308	0.430	0.366	0.246	0.345	0.118	0.176	0.192	0.130	0.439	0.161	0.138	0.158	0.285	0.271	0.431	0.263	0.212
PC 36:4	782.6/154.8	pos.	0.158	0.273	0.190	0.163	0.143	0.181	0.101	0.072	0.094	0.126	0.182	0.099	0.110	0.099	0.140	0.225	0.104	0.113
PC 36:4	782.6/147.7	pos.	0.110	0.126	0.122	0.195	0.114	0.155	0.227	0.197	0.174	0.192	0.154	0.250	0.179	0.111	0.421	0.137	0.172	0.210
PC 36:4	782.7/144.5	pos.	2.802	3.389	2.646	4.163	3.167	4.117	3.293	3.359	3.855	2.868	4.055	4.549	4.047	3.067	3.882	2.836	3.749	4.333
PC 36:3	784.7/146.3	pos.	1.574	1.444	1.550	1.420	1.353	0.772	1.487	1.028	1.290	1.871	0.840	1.352	1.071	1.430	2.235	2.336	1.555	1.324
PC 36:2	786.7/147.7	pos.	5.106	5.192	4.539	6.990	5.622	4.078	6.555	6.190	5.777	6.691	3.750	4.324	5.337	5.148	5.321	4.391	4.902	4.868
PC 36:1	788.7/148.4	pos.	1.697	1.215	1.409	1.065	1.663	1.620	1.263	1.465	1.211	1.352	1.249	1.110	1.395	1.400	1.141	1.675	1.562	1.485

PC 36:0	790.6/148.7	pos.	0.104	0.064	0.050	0.062	0.074	0.051	0.063	0.061	0.052	0.037	0.072	0.057	0.059	0.052	0.046	0.073	0.068	0.080
PC A38:6	792.7/143.1	pos.	0.028	0.029	0.023	0.029	0.021	0.042	0.091	0.044	0.031	0.047	0.044	0.085	0.048	0.082	0.049	0.060	0.078	0.076
PC A38:5	794.7/142.7	pos.	0.208	0.171	0.205	0.209	0.125	0.144	0.154	0.185	0.146	0.185	0.192	0.189	0.178	0.309	0.289	0.231	0.280	0.207
PC A38:4	796.7/143.6	pos.	0.242	0.215	0.213	0.217	0.237	0.261	0.189	0.197	0.210	0.202	0.158	0.200	0.243	0.313	0.242	0.427	0.278	0.222
PC 38:10	798.6/144.7	pos.	0.068	0.034	0.046	0.045	0.026	0.038	0.060	0.064	0.040	0.046	0.040	0.024	0.033	0.056	0.097	0.103	0.052	0.064
PC 38:9	800.6/147.1	pos.	0.083	0.104	0.083	0.042	0.072	0.047	0.048	0.041	0.042	0.073	0.052	0.031	0.030	0.051	0.120	0.087	0.030	0.030
PC 38:8	802.6/148.5	pos.	0.027	0.064	0.025	0.014	0.047	0.035	0.014	0.025	0.008	0.011	0.035	0.010	0.026	0.002	0.009	0.038	0.014	0.005
PC 38:7	804.7/142.7	pos.	0.104	0.078	0.119	0.059	0.079	0.021	0.078	0.043	0.028	0.068	0.026	0.033	0.030	0.060	0.070	0.097	0.048	0.073
PC 38:6	806.7/142.7	pos.	0.738	0.653	0.745	0.629	0.742	1.389	1.472	0.822	1.275	1.237	1.995	1.427	1.460	1.494	1.366	0.826	1.545	1.788
PC 38:5	808.6/154.1	pos.	0.054	0.055	0.068	0.024	0.053	0.036	0.021	0.014	0.017	0.055	0.044	0.017	0.024	0.019	0.018	0.052	0.019	0.024
PC 38:5	808.7/144.9	pos.	1.208	1.121	1.328	0.585	1.257	0.749	0.514	0.335	0.327	0.543	0.775	0.506	0.538	0.540	0.352	0.925	0.526	0.462
PC 38:5	808.7/142.4	pos.	2.325	1.136	1.339	0.144	1.135	0.891	1.319	0.968	0.987	1.821	0.544	1.252	1.141	1.409	1.554	2.764	1.386	1.183
PC 38:4	810.6/153.7	pos.	0.286	0.413	0.352	0.244	0.321	0.388	0.140	0.388	0.325	0.116	0.353	0.107	0.402	0.142	0.080	0.234	0.124	0.377
PC 38:4	810.7/141.9	pos.	7.655	8.446	7.900	8.988	8.949	10.078	7.024	9.254	7.239	5.203	7.466	5.949	8.462	5.630	4.056	5.380	5.269	7.711
PC 38:3	812.7/143.8	pos.	0.954	0.742	0.774	0.022	0.124	0.754	0.471	0.457	0.587	0.537	0.721	0.023	0.111	0.567	0.372	1.036	0.663	0.029
PC 38:2	814.6/145.4	pos.	0.180	0.068	0.088	0.203	0.176	0.148	0.157	0.192	0.158	0.150	0.069	0.150	0.241	0.157	0.166	0.077	0.169	0.219
PC 38:1	816.7/147.3	pos.	0.025	0.032	0.030	0.014	0.018	0.013	0.008	0.019	0.014	0.008	0.032	0.003	0.009	0.008	0.020	0.032	0.022	0.015
PC 38:0	818.7/139.5	pos.	0.016	0.020	0.033	0.014	0.016	0.030	0.026	0.019	0.037	0.050	0.055	0.041	0.032	0.089	0.036	0.016	0.061	0.049
PC A40:6	820.6/140.9	pos.	0.085	0.083	0.076	0.061	0.070	0.103	0.092	0.077	0.075	0.111	0.083	0.104	0.097	0.130	0.087	0.088	0.132	0.119
PC A40:5	822.7/142	pos.	0.087	0.084	0.039	0.042	0.046	0.033	0.060	0.024	0.041	0.039	0.047	0.055	0.055	0.086	0.092	0.072	0.071	0.049
PC A40:4	824.7/140.8	pos.	0.092	0.149	0.161	0.061	0.102	0.021	0.063	0.042	0.060	0.051	0.040	0.030	0.047	0.053	0.052	0.064	0.048	0.060
PC 40:10	826.6/145.3	pos.	0.024	0.068	0.025	0.038	0.015	0.054	0.012	0.115	0.012	0.007	0.066	0.021	0.062	0.017	0.052	0.022	0.011	0.014
PC 40:9	828.7/138.6	pos.	0.036	0.026	0.031	0.010	0.031	0.007	0.010	0.022	0.006	0.017	0.006	0.004	0.014	0.007	0.012	0.054	0.008	0.006
PC 40:8	830.7/138.3	pos.	0.334	0.221	0.323	0.266	0.309	0.109	0.091	0.048	0.066	0.105	0.077	0.048	0.202	0.073	0.215	0.496	0.082	0.063
PC 40:7	832.7/139.2	pos.	0.337	0.261	0.353	0.150	0.273	0.236	0.215	0.135	0.186	0.303	0.185	0.166	0.182	0.273	0.320	0.445	0.297	0.244
PC 40:6	834.7/139.3	pos.	1.751	1.869	1.877	1.141	1.974	4.206	2.624	2.651	2.762	2.232	4.271	2.010	3.565	2.779	1.379	0.811	2.676	3.099
PC 40:5	836.6/139.7	pos.	1.718	0.503	0.540	0.883	0.881	0.545	0.838	0.452	0.508	0.651	0.867	0.830	0.569	0.823	0.601	0.834	0.862	0.701
PC 40:4	838.6/141.1	pos.	0.550	0.510	0.685	0.265	0.563	0.226	0.265	0.133	0.163	0.218	0.003	0.220	0.241	0.158	0.283	0.327	0.185	0.229
PC 40:3	840.7/141.5	pos.	0.062	0.016	0.041	0.018	0.012	0.018	0.026	0.009	0.012	0.026	0.025	0.020	0.011	0.017	0.043	0.025	0.017	0.022
PC 40:2	842.5/138.1	pos.	0.052	0.040	0.037	0.169	0.037	0.024	0.032	0.037	0.038	0.019	0.027	0.034	0.020	0.003	0.033	0.011	0.041	0.024
PC 40:1	844.6/138	pos.	0.006	0.005	0.003	0.008	0.000	0.000	0.000	0.000	0.001	0.001	0.000	0.000	0.000	0.002	0.000	0.002	0.000	0.001
PC 40:0	846.6/138.2	pos.	0.008	0.008	0.008	0.005	0.006	0.006	0.006	0.004	0.004	0.007	0.007	0.005	0.005	0.008	0.007	0.007	0.009	0.008
PC A42:6	848.6/138.2	pos.	0.037	0.038	0.038	0.015	0.037	0.037	0.024	0.021	0.024	0.030	0.044	0.016	0.031	0.029	0.022	0.029	0.022	0.027
PC A42:5	850.6/139.9	pos.	0.032	0.029	0.039	0.010	0.025	0.008	0.009	0.007	0.007	0.009	0.008	0.008	0.011	0.010	0.014	0.020	0.010	0.008
PC 42:10	854.6/135.6	pos.	0.079	0.057	0.073	0.043	0.078	0.048	0.040	0.014	0.028	0.037	0.044	0.018	0.074	0.040	0.074	0.088	0.035	0.024
PC 42:9	856.6/136.8	pos.	0.126	0.090	0.140	0.057	0.110	0.028	0.032	0.024	0.022	0.025	0.029	0.018	0.033	0.027	0.047	0.148	0.026	0.027
PC 42:8	858.6/137.1	pos.	0.100	0.082	0.145	0.049	0.081	0.020	0.020	0.017	0.011	0.023	0.019	0.018	0.021	0.018	0.027	0.123	0.022	0.024
PC 42:7	860.6/137.5	pos.	0.046	0.044	0.071	0.009	0.031	0.016	0.010	0.009	0.012	0.016	0.016	0.014	0.014	0.013	0.017	0.037	0.014	0.017
PC 42:6	862.6/138.2	pos.	0.038	0.032	0.047	0.013	0.024	0.021	0.015	0.027	0.022	0.023	0.020	0.017	0.024	0.024	0.018	0.033	0.033	0.022

PC 42:5	864.6/138.3	pos.	0.027	0.023	0.045	0.032	0.024	0.017	0.024	0.029	0.032	0.037	0.018	0.030	0.033	0.023	0.016	0.026	0.036	0.050
PC 42:4	866.6/137.2	pos.	0.013	0.008	0.005	0.024	0.010	0.005	0.005	0.007	0.015	0.007	0.007	0.009	0.002	0.001	0.007	0.003	0.012	0.000
PC 42:3	868.5/138.2	pos.	0.011	0.008	0.009	0.018	0.006	0.003	0.004	0.004	0.001	0.003	0.003	0.004	0.002	0.002	0.005	0.002	0.004	0.002
PC 44:12	878.6/134	pos.	0.009	0.007	0.006	0.006	0.009	0.008	0.010	0.004	0.006	0.007	0.007	0.008	0.011	0.013	0.014	0.007	0.012	0.007
PC 44:11	880.6/135.2	pos.	0.019	0.014	0.020	0.010	0.019	0.007	0.006	0.002	0.004	0.007	0.007	0.003	0.006	0.006	0.008	0.014	0.007	0.004
PC 44:10	882.6/135.4	pos.	0.027	0.017	0.031	0.006	0.021	0.006	0.007	0.003	0.003	0.007	0.005	0.004	0.003	0.005	0.006	0.019	0.005	0.006
PC 44:9	884.6/136.3	pos.	0.015	0.009	0.018	0.004	0.011	0.002	0.003	0.003	0.002	0.003	0.003	0.004	0.003	0.004	0.003	0.015	0.003	0.004
PC 44:7	888.6/136.4	pos.	0.006	0.003	0.006	0.007	0.004	0.007	0.010	0.006	0.005	0.004	0.004	0.005	0.006	0.005	0.004	0.004	0.004	0.007
PC 44:5	892.6/135.3	pos.	0.006	0.002	0.003	0.003	0.006	0.002	0.003	0.003	0.003	0.004	0.002	0.003	0.004	0.003	0.005	0.003	0.004	0.004
PC 46:9	912.5/137.2	pos.	0.003	0.002	0.001	0.002	0.003	0.005	0.007	0.005	0.004	0.004	0.006	0.004	0.005	0.004	0.003	0.001	0.005	0.006
PC 46:8	914.6/136.8	pos.	0.002	0.002	0.001	0.002	0.001	0.002	0.003	0.003	0.003	0.002	0.001	0.003	0.003	0.003	0.002	0.000	0.003	0.004
SM d18:1;14:1	673.5/178.3	pos.	0.001	0.001	0.001	0.001	0.001	0.000	0.000	0.001	0.000	0.000	0.000	0.001	0.000	0.001	0.001	0.001	0.001	0.000
SM d18:1;14:0	675.6/177.7	pos.	0.012	0.017	0.018	0.011	0.013	0.014	0.018	0.017	0.010	0.012	0.015	0.023	0.013	0.018	0.026	0.043	0.022	0.010
SM d18:0;14:0	677.7/177.7	pos.	0.001	0.001	0.001	0.001	0.001	0.001	0.000	0.001	0.000	0.000	0.000	0.000	0.000	0.000	0.002	0.002	0.000	0.000
SM d18:1;16:1	701.7/176.3	pos.	0.023	0.028	0.028	0.037	0.023	0.022	0.027	0.025	0.020	0.018	0.025	0.023	0.036	0.026	0.044	0.048	0.025	0.015
SM d18:1;16:0	703.6/176	pos.	0.863	0.834	1.027	1.007	0.758	1.091	1.204	1.063	0.930	0.976	1.158	1.236	1.188	1.165	1.260	1.431	1.080	1.065
SM d18:0;16:0	705.6/175.7	pos.	0.036	0.038	0.038	0.071	0.043	0.021	0.055	0.043	0.039	0.041	0.046	0.051	0.048	0.065	0.101	0.088	0.062	0.050
SM d18:1;18:3	725.6/178.2	pos.	0.011	0.013	0.013	0.016	0.013	0.015	0.022	0.019	0.011	0.013	0.013	0.018	0.017	0.018	0.014	0.013	0.016	0.021
SM d18:1;18:2	727.7/174.3	pos.	0.000	0.000	0.001	0.002	0.001	0.001	0.002	0.001	0.001	0.001	0.001	0.001	0.000	0.004	0.002	0.003	0.001	0.002
SM d18:1;18:1	729.7/174.5	pos.	0.023	0.023	0.022	0.048	0.018	0.023	0.024	0.027	0.025	0.017	0.021	0.027	0.031	0.035	0.037	0.056	0.027	0.015
SM d18:1;18:0	731.7/174.5	pos.	0.568	0.310	0.307	0.597	0.305	0.567	0.375	0.550	0.537	0.550	0.428	0.531	0.492	0.794	0.403	0.489	0.599	0.658
SM d18:0;18:0	733.6/174.3	pos.	0.000	0.016	0.013	0.084	0.016	0.024	0.024	0.027	0.012	0.004	0.021	0.024	0.020	0.033	0.035	0.022	0.036	0.017
SM d18:1;20:5	749.6/177.8	pos.	0.001	0.001	0.001	0.002	0.002	0.002	0.002	0.002	0.005	0.002	0.002	0.002	0.002	0.002	0.003	0.003	0.002	0.001
SM d18:1;20:1	757.7/173	pos.	0.027	0.025	0.027	0.017	0.020	0.013	0.015	0.014	0.013	0.009	0.012	0.016	0.012	0.022	0.022	0.025	0.021	0.010
SM d18:1;20:0	759.7/173.4	pos.	0.233	0.235	0.255	0.154	0.209	0.168	0.153	0.198	0.161	0.200	0.177	0.237	0.172	0.238	0.213	0.180	0.258	0.194
SM d18:0;20:0	761.7/173.2	pos.	0.043	0.037	0.016	0.019	0.013	0.014	0.010	0.009	0.017	0.036	0.013	0.017	0.011	0.045	0.023	0.014	0.023	0.010
SM d18:1;22:3	781.5/177	pos.	0.004	0.003	0.004	0.004	0.005	0.006	0.008	0.005	0.005	0.005	0.003	0.005	0.006	0.006	0.003	0.002	0.004	0.009
SM d18:1;22:2	783.7/172.2	pos.	0.009	0.010	0.008	0.010	0.007	0.007	0.004	0.005	0.005	0.008	0.011	0.008	0.006	0.007	0.009	0.012	0.007	0.008
SM d18:1;22:1	785.7/172	pos.	0.050	0.050	0.051	0.036	0.045	0.045	0.038	0.037	0.036	0.035	0.034	0.042	0.035	0.041	0.066	0.067	0.048	0.028
SM d18:1;22:0	787.7/172.6	pos.	0.386	0.379	0.401	0.318	0.335	0.563	0.387	0.408	0.471	0.386	0.420	0.352	0.388	0.367	0.425	0.415	0.381	0.329
SM d18:0;22:0	789.7/172.6	pos.	0.028	0.025	0.027	0.029	0.025	0.028	0.025	0.023	0.013	0.021	0.024	0.021	0.025	0.021	0.043	0.023	0.019	0.020
SM d18:1;24:6	803.7/172.3	pos.	0.022	0.019	0.031	0.035	0.025	0.010	0.031	0.014	0.014	0.017	0.014	0.005	0.017	0.022	0.030	0.028	0.012	0.012
SM d18:1;24:5	805.7/173.6	pos.	0.001	0.000	0.000	0.000	0.002	0.002	0.001	0.001	0.007	0.004	0.002	0.003	0.001	0.000	0.003	0.000	0.001	0.002
SM d18:1;24:3	809.7/171.4	pos.	0.014	0.013	0.010	0.011	0.008	0.008	0.011	0.010	0.007	0.007	0.007	0.013	0.009	0.007	0.008	0.015	0.011	0.013
SM d18:1;24:2	811.7/171.2	pos.	0.102	0.116	0.125	0.096	0.086	0.088	0.077	0.071	0.063	0.063	0.081	0.106	0.079	0.078	0.092	0.091	0.091	0.073
SM d18:1;24:1	813.7/171.2	pos.	0.605	0.633	0.722	0.533	0.515	0.671	0.510	0.510	0.505	0.511	0.602	0.768	0.560	0.698	0.622	0.476	0.685	0.553
SM d18:1;24:0	815.6/171.8	pos.	0.437	0.409	0.480	0.399	0.388	0.449	0.368	0.409	0.392	0.370	0.395	0.311	0.424	0.437	0.403	0.389	0.377	0.366
SM d18:0;24:0	817.6/172.1	pos.	0.021	0.016	0.027	0.024	0.028	0.021	0.014	0.017	0.016	0.022	0.022	0.016	0.020	0.019	0.027	0.029	0.019	0.018
SM d18:1;26:6	831.6/173.6	pos.	0.010	0.017	0.014	0.004	0.013	0.005	0.010	0.018	0.019	0.012	0.010	0.011	0.012	0.012	0.019	0.014	0.001	0.005

SM d18:1;26:5	833.7/174.3	pos.	0.007	0.006	0.008	0.008	0.011	0.008	0.008	0.009	0.016	0.010	0.009	0.007	0.007	0.005	0.016	0.016	0.007	0.008
SM d18:1;26:4	835.7/169.7	pos.	0.008	0.009	0.009	0.009	0.008	0.008	0.008	0.009	0.008	0.009	0.008	0.010	0.009	0.010	0.009	0.005	0.008	0.012
SM d18:1;26:2	839.6/170.1	pos.	0.009	0.009	0.010	0.006	0.008	0.006	0.008	0.005	0.006	0.007	0.005	0.010	0.006	0.009	0.005	0.005	0.011	0.008
SM d18:1;26:1	841.6/170.4	pos.	0.015	0.026	0.049	0.039	0.051	0.046	0.053	0.032	0.028	0.027	0.022	0.080	0.048	0.033	0.015	0.031	0.080	0.046
SM d18:1;26:0	843.6/172.5	pos.	0.014	0.012	0.017	0.021	0.015	0.009	0.010	0.017	0.013	0.011	0.008	0.032	0.014	0.028	0.009	0.009	0.032	0.009
lysoPC C16:1	494.5/197.8	pos.	0.010	0.005	0.010	0.007	0.004	0.002	0.001	0.002	0.002	0.003	0.003	0.002	0.003	0.004	0.003	0.009	0.003	0.002
lysoPC C16:0	496.5/195.9	pos.	0.145	0.113	0.170	0.227	0.104	0.245	0.112	0.156	0.179	0.086	0.218	0.196	0.351	0.151	0.162	0.152	0.185	0.107
lysoPC C18:3	518.5/196.1	pos.	0.041	0.029	0.042	0.066	0.029	0.068	0.024	0.032	0.056	0.024	0.062	0.043	0.103	0.036	0.036	0.050	0.056	0.024
lysoPC C18:2	520.5/186.8	pos.	0.066	0.021	0.069	0.067	0.046	0.016	0.052	0.018	0.059	0.057	0.018	0.005	0.082	0.017	0.018	0.029	0.017	0.013
lysoPC C18:2	520.5/194.7	pos.	0.029	0.010	0.023	0.028	0.018	0.009	0.018	0.016	0.019	0.021	0.010	0.008	0.038	0.010	0.013	0.022	0.011	0.009
lysoPC C18:1	522.5/192.2	pos.	0.046	0.020	0.047	0.038	0.029	0.023	0.017	0.025	0.024	0.020	0.016	0.017	0.047	0.020	0.025	0.066	0.025	0.013
lysoPC C18:1	522.5/184.8	pos.	0.053	0.011	0.047	0.027	0.031	0.011	0.020	0.008	0.027	0.032	0.010	0.003	0.044	0.009	0.010	0.031	0.010	0.006
lysoPC C18:0	524.5/190.6	pos.	0.432	0.308	0.413	0.595	0.345	0.513	0.202	0.404	0.321	0.158	0.316	0.194	0.835	0.223	0.155	0.258	0.261	0.174
lysoPC C20:5	542.5/186.6	pos.	0.028	0.007	0.029	0.029	0.019	0.003	0.017	0.005	0.027	0.026	0.005	0.002	0.039	0.006	0.005	0.015	0.006	0.003
lysoPC C20:4	544.5/183	pos.	0.214	0.055	0.209	0.147	0.139	0.074	0.066	0.032	0.117	0.057	0.067	0.013	0.230	0.029	0.013	0.065	0.030	0.029
lysoPC C20:4	544.5/190.6	pos.	0.045	0.012	0.006	0.037	0.025	0.023	0.016	0.022	0.024	0.013	0.015	0.006	0.072	0.013	0.008	0.028	0.011	0.009
lysoPC C20:3	546.5/190.7	pos.	0.118	0.080	0.134	0.192	0.078	0.177	0.049	0.133	0.091	0.035	0.099	0.054	0.290	0.069	0.042	0.075	0.066	0.031
lysoPC C20:2	548.5/190.5	pos.	0.005	0.005	0.005	0.003	0.003	0.003	0.002	0.003	0.003	0.002	0.003	0.004	0.005	0.001	0.001	0.004	0.003	0.002
lysoPC C20:1	550.6/187.9	pos.	0.002	0.001	0.002	0.001	0.001	0.001	0.000	0.001	0.001	0.000	0.001	0.001	0.001	0.001	0.001	0.001	0.001	0.000
