# Supplementary material for: Aberrant hepatic lipid storage and metabolism in canine portosystemic shunts
Source: PLoS One. 2017 Oct 19;12(10):e0186491. doi: 10.1371/journal.pone.0186491 (PMC5648188; doi:10.1371/journal.pone.0186491)
Supplement: S4 Table — Only TAG species contributing more than 0.015% on average are included. (PDF) [file pone.0186491.s009.pdf]

	mz/Rt	CTR_10	CTR_12	CTR_8	CTR_9	ExH_13	ExH_14	ExH_15	ExH_1	ExH_2	ExH_3	InH_17	InH_18	InH_4	InH_5	InH_6	InH_7
TAG 48:1	805.7/330	0.13	0.18	0.23	0.16	0.50	0.26	0.33	0.36	0.26	0.95	0.54	0.37	1.15	0.32	0.45	0.26
TAG 49:1	819.8/339	0.10	0.28	0.38	0.08	0.15	0.05	0.04	0.09	0.09	0.21	0.12	0.06	0.15	0.06	0.17	0.11
TAG 50:1	833.8/350	0.48	0.56	0.68	0.38	1.72	0.98	1.16	1.31	0.97	2.12	1.66	1.28	2.70	1.38	1.52	1.22
TAG 51:1	847.8/360	0.03	0.06	0.12	0.03	0.18	0.09	0.05	0.10	0.09	0.18	0.15	0.08	0.15	0.07	0.22	0.18
TAG 52:1	861.7/373	0.56	0.62	0.89	0.30	1.23	0.66	0.44	0.56	0.70	1.28	1.06	0.69	1.33	0.63	1.36	1.20
TAG 48:2	803.8/316	0.13	0.14	0.15	0.24	0.31	0.23	0.25	0.25	0.18	0.62	0.40	0.31	0.85	0.22	0.28	0.17
TAG 50:2	831.7/335	0.96	1.03	0.94	1.09	1.71	1.39	1.18	1.26	1.18	2.38	1.75	1.94	3.12	1.25	1.61	1.15
TAG 51:2	845.8/345	0.14	0.15	0.20	0.11	0.30	0.23	0.09	0.18	0.16	0.30	0.25	0.18	0.29	0.13	0.32	0.25
TAG 52:2	859.8/356	5.16	5.53	5.16	3.79	7.29	6.66	4.38	4.89	5.98	6.65	5.82	6.95	7.62	5.10	7.40	7.36
TAG 53:2	873.7/367	0.19	0.22	0.30	0.10	0.33	0.30	0.10	0.17	0.24	0.27	0.25	0.19	0.22	0.15	0.42	0.43
TAG 54:2	887.8/380	2.68	2.72	3.11	1.44	1.09	1.32	0.43	0.61	2.20	1.31	1.27	1.22	1.48	0.59	1.88	2.01
TAG 56:2	915.7/408	0.21	0.21	0.30	0.11	0.06	0.07	0.02	0.04	0.18	0.05	0.09	0.05	0.09	0.03	0.12	0.20
TAG 48:3	801.7/304	0.17	0.17	0.15	0.41	0.48	0.35	0.62	0.49	0.33	0.94	0.63	0.37	1.06	0.53	0.35	0.23
TAG 50:3	829.8/320	1.48	1.52	1.18	3.07	2.89	2.45	2.54	2.43	2.33	4.73	3.06	3.56	4.96	2.42	2.46	1.56
TAG 51:3	843.8/329	0.25	0.27	0.32	0.30	0.66	0.48	0.30	0.45	0.39	0.73	0.51	0.46	0.60	0.38	0.56	0.46
TAG 52:3	857.8/339	11.90	12.91	10.70	13.27	24.76	21.44	20.49	17.68	22.01	23.96	17.54	23.94	22.37	22.70	19.42	21.22
TAG 53:3	871.8/349	0.48	0.58	0.68	0.35	0.98	0.90	0.39	0.52	0.64	0.94	0.78	0.63	0.76	0.55	1.10	1.18
TAG 53:3	871.8/338	0.28	0.32	0.26	0.33	0.61	0.55	0.54	0.46	0.51	0.58	0.48	0.66	0.59	0.55	0.46	0.52
TAG 54:3	885.8/361	13.98	13.28	14.29	7.77	4.96	7.05	2.52	2.99	9.74	5.84	5.48	6.21	6.57	3.43	7.01	8.44
TAG 56:3	913.8/386	1.04	0.91	1.01	0.53	0.24	0.38	0.10	0.17	0.56	0.22	0.43	0.28	0.36	0.15	0.46	0.82
TAG 58:3	941.8/415	0.20	0.21	0.28	0.11	0.05	0.06	0.02	0.03	0.14	0.03	0.06	0.04	0.07	0.02	0.07	0.14
TAG 50:4	827.7/307	0.69	0.71	0.66	2.11	1.01	0.97	1.26	1.49	0.91	1.80	1.38	1.24	1.56	1.09	0.94	0.52
TAG 51:4	841.8/315	0.14	0.15	0.18	0.27	0.28	0.23	0.21	0.33	0.18	0.36	0.27	0.23	0.29	0.23	0.28	0.19
TAG 52:4	855.8/323	7.70	8.40	6.11	15.01	15.91	14.31	21.24	14.42	15.39	14.87	12.49	15.48	12.54	19.71	11.91	11.42
TAG 53:4	869.8/334	0.41	0.50	0.60	0.46	0.78	0.74	0.47	0.58	0.58	0.78	0.73	0.62	0.72	0.57	0.83	0.78
TAG 54:4	883.8/345	19.84	18.17	16.45	11.83	6.86	12.59	6.34	4.74	12.42	8.30	8.63	10.67	9.51	6.95	8.93	12.46
TAG 55:4	897.7/356	0.53	0.53	0.60	0.35	0.67	0.63	0.37	0.41	0.49	0.55	0.61	0.58	0.63	0.43	0.72	0.81
TAG 56:4	911.8/367	1.90	1.59	1.61	1.01	0.68	0.95	0.43	0.71	1.07	0.59	1.37	0.78	0.83	0.55	1.13	1.68
TAG 57:4	925.8/380	0.22	0.23	0.28	0.12	0.11	0.11	0.04	0.06	0.17	0.10	0.13	0.11	0.12	0.06	0.19	0.20
TAG 58:4	939.8/393	0.29	0.25	0.33	0.14	0.09	0.09	0.04	0.06	0.16	0.06	0.17	0.11	0.16	0.05	0.16	0.23
TAG 60:4	967.8/422	0.12	0.11	0.14	0.05	0.03	0.03	0.01	0.01	0.07	0.01	0.03	0.04	0.05	0.01	0.04	0.08
TAG 52:5	853.8/311	1.85	2.01	1.65	6.12	3.26	2.86	4.81	4.49	2.57	3.24	2.86	2.81	2.27	4.59	2.69	1.24
TAG 54:5	881.8/329	11.53	9.43	8.04	9.36	4.74	7.73	6.96	9.57	7.26	5.25	6.11	6.42	5.13	6.34	5.68	7.39
TAG 55:5	895.7/339	0.45	0.47	0.66	0.46	0.71	0.65	0.59	0.53	0.59	0.66	0.63	0.72	0.67	0.63	0.68	0.70
TAG 56:5	909.7/350	1.92	2.45	3.28	1.71	2.58	2.31	2.76	3.29	1.68	0.86	3.86	2.18	1.80	2.75	3.25	2.45
TAG 57:5	923.8/362	0.52	0.53	0.59	0.29	0.20	0.28	0.12	0.14	0.32	0.20	0.23	0.23	0.22	0.13	0.29	0.37
TAG 58:5	937.8/374	0.43	0.34	0.53	0.23	0.25	0.24	0.19	0.20	0.23	0.19	0.51	0.31	0.43	0.17	0.39	0.50
TAG 60:5	965.8/399	0.11	0.08	0.07	0.04	0.03	0.03	0.02	0.02	0.05	0.03	0.08	0.04	0.09	0.01	0.07	0.07
TAG 55:6	893.7/324	0.28	0.35	0.55	0.39	0.45	0.36	0.39	0.62	0.45	0.27	0.51	0.36	0.27	0.41	0.53	0.32
TAG 56:6	907.7/335	3.00	3.38	4.42	3.43	3.65	2.57	5.29	6.57	1.88	2.72	5.01	2.52	2.03	4.96	4.57	2.86
TAG 57:6	921.7/345	0.40	0.38	0.41	0.25	0.18	0.27	0.17	0.17	0.22	0.18	0.23	0.23	0.18	0.16	0.26	0.34
TAG 58:6	935.7/356	0.75	0.60	0.87	0.46	0.49	0.48	0.54	0.49	0.35	0.44	0.95	0.51	0.55	0.40	0.73	1.01
TAG 59:6	949.8/367	0.04	0.04	0.06	0.03	0.03	0.02	0.01	0.01	0.02	0.01	0.04	0.02	0.02	0.02	0.03	0.06
TAG 60:6	963.8/379	0.10	0.07	0.06	0.04	0.06	0.05	0.03	0.02	0.03	0.06	0.17	0.10	0.16	0.03	0.10	0.11
TAG 54:7	877.7/307	0.78	1.04	0.99	3.27	0.81	0.92	2.19	2.22	0.69	0.76	1.02	0.32	0.45	1.22	0.95	0.11
TAG 56:7	905.7/326	1.74	1.97	2.78	3.90	1.85	2.28	3.01	3.94	1.62	1.19	2.93	1.27	0.91	2.74	2.18	1.55
TAG 57:7	919.7/329	0.17	0.16	0.22	0.15	0.09	0.13	0.19	0.12	0.10	0.08	0.13	0.10	0.07	0.18	0.22	0.12
TAG 58:7	933.7/340	0.72	0.67	0.95	0.56	0.85	0.51	0.74	0.64	0.32	0.45	0.92	0.42	0.42	0.84	1.11	0.95
TAG 60:7	961.7/361	0.07	0.04	0.09	0.04	0.09	0.06	0.05	0.05	0.04	0.09	0.27	0.12	0.17	0.04	0.14	0.11
TAG 56:8	903.7/312	0.84	1.26	1.67	1.72	1.09	0.79	2.35	2.96	0.51	0.72	1.63	0.72	0.41	1.83	1.04	0.77
TAG 58:8	931.7/334	0.44	0.59	1.00	0.49	0.53	0.25	0.67	1.30	0.20	0.29	1.06	0.39	0.24	0.62	0.64	0.44
TAG 60:8	959.7/352	0.17	0.11	0.19	0.11	0.12	0.07	0.16	0.14	0.05	0.02	0.28	0.11	0.09	0.10	0.16	0.18
TAG 58:9	929.7/318	0.55	0.65	1.11	0.77	0.35	0.27	0.84	1.64	0.18	0.20	0.80	0.28	0.19	0.56	0.50	0.32
TAG 60:9	957.7/335	0.23	0.20	0.39	0.20	0.16	0.09	0.26	0.43	0.07	0.12	0.41	0.09	0.11	0.16	0.28	0.17
TAG 58:10	927.7/309	0.28	0.37	0.64	0.46	0.25	0.14	0.67	1.66	0.13	0.13	0.59	0.18	0.11	0.43	0.33	0.18
TAG 60:10	955.7/324	0.19	0.22	0.37	0.16	0.17	0.08	0.36	0.57	0.06	0.08	0.39	0.13	0.08	0.23	0.26	0.15
TAG 62:11	981.7/327	0.02	0.01	0.03	0.01	0.03	0.01	0.05	0.05	0.01	0.02	0.10	0.02	0.02	0.02	0.05	0.02
TAG 60:12	951.7/302	0.04	0.06	0.09	0.03	0.07	0.03	0.16	0.28	0.04	0.04	0.13	0.06	0.03	0.10	0.10	0.04
